# Discovery of an Antarctic Ascidian-Associated Uncultivated *Verrucomicrobia* with Antimelanoma Palmerolide Biosynthetic Potential

**DOI:** 10.1128/mSphere.00759-21

**Published:** 2021-12-01

**Authors:** Alison E. Murray, Chien-Chi Lo, Hajnalka E. Daligault, Nicole E. Avalon, Robert W. Read, Karen W. Davenport, Mary L. Higham, Yuliya Kunde, Armand E. K. Dichosa, Bill J. Baker, Patrick S. G. Chain

**Affiliations:** a Division of Earth and Ecosystem Science, Desert Research Institute, Reno, Nevada, USA; b Bioscience Division, Los Alamos National Laboratory, Los Alamos, New Mexico, USA; c Department of Chemistry, University of South Florida, Tampa, Florida, USA; Clemson University

**Keywords:** Antarctic, Synoicihabitans palmerolidicus, *Synoicum adareanum*, *Verrucomicrobia*, anticancer, ascidian natural product, biosynthetic gene cluster, microbiome metagenome, palmerolide, secondary metabolite

## Abstract

The Antarctic marine ecosystem harbors a wealth of biological and chemical innovation that has risen in concert over millennia since the isolation of the continent and formation of the Antarctic circumpolar current. Scientific inquiry into the novelty of marine natural products produced by Antarctic benthic invertebrates led to the discovery of a bioactive macrolide, palmerolide A, that has specific activity against melanoma and holds considerable promise as an anticancer therapeutic. While this compound was isolated from the Antarctic ascidian Synoicum adareanum, its biosynthesis has since been hypothesized to be microbially mediated, given structural similarities to microbially produced hybrid nonribosomal peptide-polyketide macrolides. Here, we describe a metagenome-enabled investigation aimed at identifying the biosynthetic gene cluster (BGC) and palmerolide A-producing organism. A 74-kbp candidate BGC encoding the multimodular enzymatic machinery (hybrid type I-*trans*-AT polyketide synthase-nonribosomal peptide synthetase and tailoring functional domains) was identified and found to harbor key features predicted as necessary for palmerolide A biosynthesis. Surveys of ascidian microbiome samples targeting the candidate BGC revealed a high correlation between palmerolide gene targets and a single 16S rRNA gene variant (*R* = 0.83 to 0.99). Through repeated rounds of metagenome sequencing followed by binning contigs into metagenome-assembled genomes, we were able to retrieve a nearly complete genome (10 contigs) of the BGC-producing organism, a novel verrucomicrobium within the *Opitutaceae* family that we propose here as “*Candidatus* Synoicihabitans palmerolidicus.” The refined genome assembly harbors five highly similar BGC copies, along with structural and functional features that shed light on the host-associated nature of this unique bacterium.

**IMPORTANCE** Palmerolide A has potential as a chemotherapeutic agent to target melanoma. We interrogated the microbiome of the Antarctic ascidian, Synoicum adareanum, using a cultivation-independent high-throughput sequencing and bioinformatic strategy. The metagenome-encoded biosynthetic machinery predicted to produce palmerolide A was found to be associated with the genome of a member of the S. adareanum core microbiome. Phylogenomic analysis suggests the organism represents a new deeply branching genus, “*Candidatus* Synoicihabitans palmerolidicus,” in the *Opitutaceae* family of the *Verrucomicrobia* phylum. The *Ca*. Synoicihabitans palmerolidicus 4.29-Mb genome encodes a repertoire of carbohydrate-utilizing and transport pathways, a chemotaxis system, flagellar biosynthetic capacity, and other regulatory elements enabling its ascidian-associated lifestyle. The palmerolide producer’s genome also contains five distinct copies of the large palmerolide biosynthetic gene cluster that may provide structural complexity of palmerolide variants.

## INTRODUCTION

Across the world’s oceans, marine benthic invertebrates harbor a rich source of natural products that serve metabolic and ecological roles *in situ*. These compounds provide a multitude of medicinal and biotechnological applications to science, health, and industry. The organisms responsible for their biosynthesis are often unknown (1, 2). Increasingly, these metabolites, especially in the polyketide class (*trans*-AT in particular), are found to be produced by microbial counterparts associated with the invertebrate host (3–5). Invertebrates, including sponges, corals, and ascidians for example, harbor a wealth of diverse microbes, few of which have been cultivated (e.g., references [Bibr B6][Bibr B7][Bibr B8]). Genomic tools, in particular, are revealing biochemical pathways potentially critical in the host-microbe associations (9). Microbes that form persistent mutualistic (symbiotic) associations provide key roles in host ecology, such as provision of metabolic requirements, production of adaptive features such as photoprotective pigments, bioluminescence, or antifoulants, and biosynthesis of chemical defense agents.

Antarctic marine ecosystems harbor species-rich macrobenthic communities (10–12), which have been the subject of natural product investigations over the past 30 years, resulting in the identification of >600 metabolites (13). Initially, it was not known whether the same selective pressures (namely, predation and competition, e.g., reference 14) that operate in mid and low latitudes would drive benthic organisms at the poles to create novel chemistry (15). However, this does appear to be the case, and novel natural products have been discovered across algae, sponges, corals, nudibranchs, echinoderms, bryozoans, ascidians, and increasingly among microorganisms (16) for which the ecological roles have been deduced in a number of cases (13, 17). Studies of Antarctic benthic invertebrate-microbe associations, however, pale in comparison to studies at lower latitudes, yet the few studies that have been reported suggest these associations (i) harbor an untapped reservoir of biological diversity (18–21) including fungi (22), (ii) are host species specific (23, 24), (iii) provide the host with sources of nitrogen and fixed carbon (25), and (iv) have biosynthetic functional potential (26, 27).

**FIG 1:**
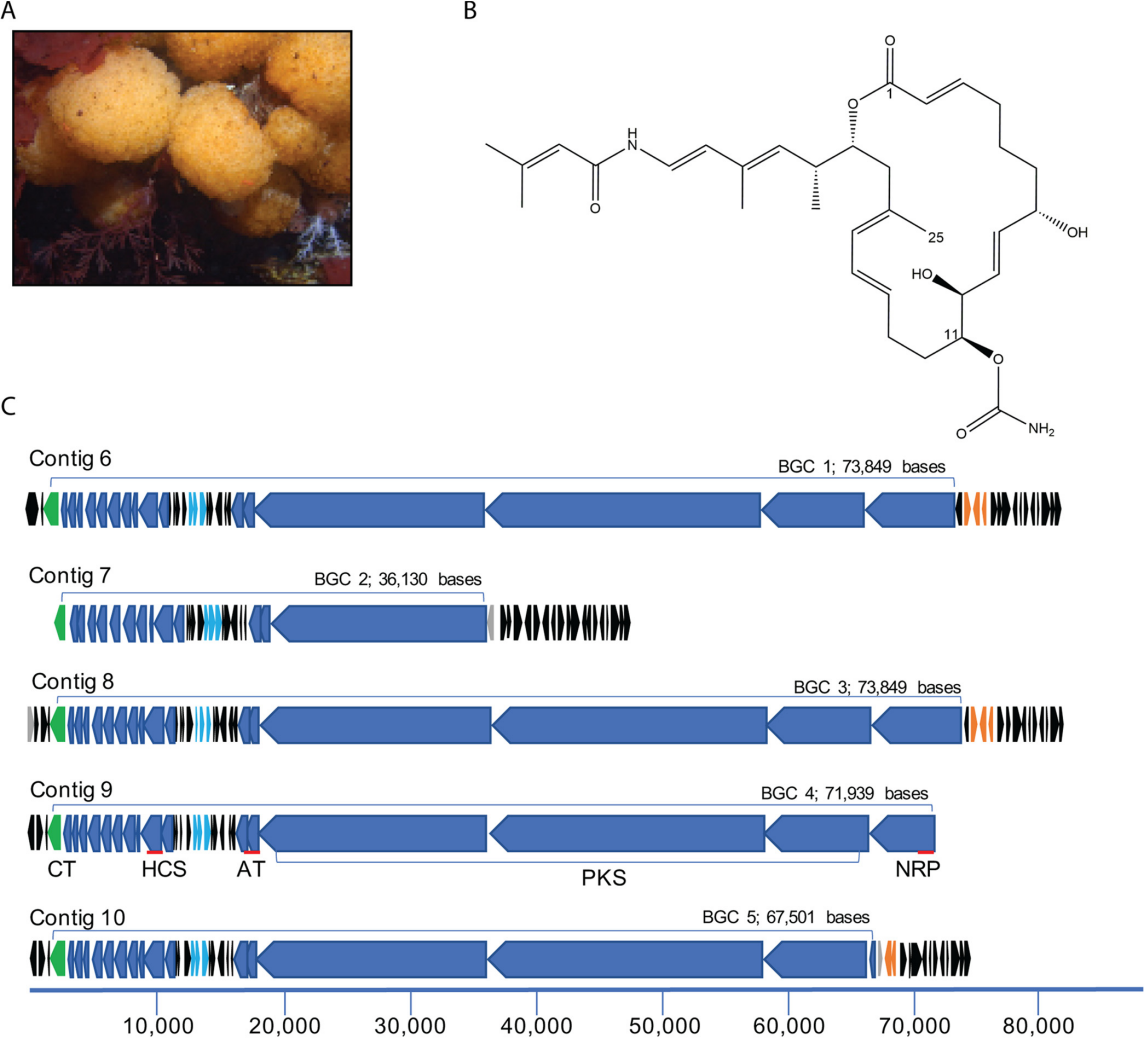
Palmerolide A, a cytotoxic macrolide with antimelanoma activity, is found in the tissues of *Synoicum adareanum* in which a candidate biosynthetic gene cluster has been identified. (A) *S. adareanum* occurs on rocky coastal seafloor habitats in the Antarctic; this study focused on the region offshore of Anvers Island in the Antarctic Peninsula. (B) Palmerolide A is the product of a hybrid PKS-NRPS system in which biosynthesis begins with a PKS starter unit followed by incorporation of a glycine subunit by an NRPS module. Subsequent elongation, cyclization, and termination steps follow. Two additional features of the molecule include a methyl group on C-25 and a carbamate group on C-11. (C) Five repeats encompassing candidate palmerolide biosynthetic gene clusters were identified. The BGC (in blue) is defined as starting with the NRP unit and ending at the carbamoyltransferase (green). Candidate palmerolide A biosynthetic gene cluster BGC 4 was identified from initial metagenome library assemblies. The other four clusters were identified following a third round of sequencing, assembly, and manual finishing. Primary BGC coding sequences (CDSs) and a conserved tailoring cassette are in blue. Light blue CDS are an ATP transporter with homology to an antibiotic transporter, SyrD. All black CDSs are repeated among the BGCs. Orange CDSs are transposase/integrase domains. Gray CDSs are unique, nonrepeated; and in BGC 2 and 5, the unique CDSs encode transposases, distinct from the predicted amino acid sequences of those in orange. The red lines associated with contig 9 indicate targeted quantitative PCR regions.

This study was specifically motivated by our desire to understand the biosynthetic origins of a natural product, palmerolide A, given its potent anticancer activity (28), that is found to be associated with the polyclinid Antarctic ascidian, Synoicum adareanum (Fig. 1A and B). Ascidians are known to be rich sources of bioactive natural products (9). They have been found to harbor polyketide, terpenoid, peptide, alkaloid and a few other classes of natural products, of which the majority have cytotoxic and/or antimicrobial activities. In addition to palmerolide A, a few other natural products derived from Antarctic ascidians have been reported (29–31). Ascidian-associated microbes responsible for natural product biosynthesis have been shown to be affiliated with bacterial phyla, including *Actinobacteria* (which dominates the recognized diversity), *Cyanobacteria*, *Firmicutes*, *Proteobacteria* (both *Alphaproteobacteria* and *Gammaproteobacteria*) and *Verrucomicrobia* in addition to many fungi (32, 33). Metagenome-enabled studies have been key in linking natural products to the organisms producing them in a number of cases, e.g., patellamide A and C to *Cyanobacteria*-affiliated *Prochloron* spp. (4), the tetrahydroisoquinoline alkaloid ET-743 to *Gammaproteobacteria*-affiliated *Candidatus* Endoecteinascidia frumentensis (34), patellazoles to *Alphaproteobacteria*-affiliated *Candidatus* Endolissoclinum faulkneri (35), and mandelalides to *Verrucomicrobia*-affiliated *Candidatus* Didemnitutus mandela (36). However, this is most certainly an underrepresentation of the diversity of ascidian-associated microorganisms with capabilities for synthesizing bioactive compounds, given the breadth of ascidian biodiversity (37). These linkages have yet to be investigated for Antarctic ascidians.

Palmerolide A has anticancer properties with selective activity against melanoma when tested in the National Cancer Institute 60-cell-line panel (28). This result is of particular interest, as there are few natural product therapeutics for this devastating form of cancer. Palmerolide A inhibits vacuolar ATPases, which are highly expressed in metastatic melanoma. Given the current level of understanding that macrolides often have microbial biosynthetic origins, that the holobiont metagenome has biosynthetic potential (26), and that a diverse, yet persistent core microbiome is found in palmerolide A-containing S. adareanum (27), we have hypothesized that a microbe associated with *S. adareanum* is responsible for the biosynthesis of palmerolide A.

The core microbiome of the palmerolide A-producing ascidian *S. adareanum* in samples collected across the Anvers Island archipelago (*n* = 63 samples) (27) is comprised of five bacterial phyla, including *Proteobacteria* (dominating the microbiome), *Bacteroidetes*, *Nitrospirae*, *Actinobacteria*, and *Verrucomicrobia*. A few candidate taxa in particular, were suggested to be likely palmerolide A producers based on relative abundance and biosynthetic potential determined by analysis of lineage-targeted biosynthetic capability (*Microbulbifer*, *Pseudovibrio*, *Hoeflea* genera and the family *Opitutaceae* (27). This motivated interrogation of the *S. adareanum* microbiome metagenome, with the goals of determining the metagenome-encoded biosynthetic potential, identifying candidate palmerolide A biosynthetic gene cluster(s) [BGC(s)] and establishing the identity of the palmerolide A-producing organism.

## RESULTS AND DISCUSSION

### Identification of a candidate palmerolide A biosynthetic gene cluster.

Microbe-enriched fractions of *S. adareanum* metagenomic DNA sequence from 454 and Ion Proton next-generation sequencing (NGS) libraries (almost 18 billion bases in all) were assembled independently and then merged, resulting in ∼145 Mbp of assembled bases distributed over 86,387 contigs (referred to as CoAssembly 1; see Table S1 in the supplemental material). As the metagenome sequencing effort was focused on identifying potential BGCs encoding the machinery to synthesize palmerolide A, the initial steps of analysis specifically targeted those contigs in the assembly that were >40 kbp (102 contigs representing 0.12% of all contigs, or 7.07% of all reads mapped), as the size of the macrolide ring with 24 carbons would require a large number of polyketide modules to be encoded. This large fragment subset of CoAssembly 1 was submitted to antiSMASH v.3 (38) and more recently to v.5 (39). The results indicated a heterogeneous suite of BGCs, including a bacteriocin, two nonribosomal peptide synthetases (NRPS), two hybrid NRPS-type I PKS, two terpenes, and three *trans*-AT-PKS hybrid NRPS clusters (Table S2).

10.1128/mSphere.00759-21.5 TABLE S1 Metagenome sequencing metadata for *S. adareanum* microbiome. Host lobe identities indicate sample site (Nor, Norsel; Bon, Bonaparte Point; Del, DeLaca Island) (followed by lobe designation and year sampled). Assembly 1 joined two data sets using mega-merged contigs from 454 and Ion Proton sequence data sets. Assembly 2 included Assembly 1 and PacBio assemblies in addition to the manually assembled *Ca.* Synoicihabitans palmerolidicus MAG. Download Table S1, PDF file, 0.02 MB. Copyright © 2021 Murray et al. 2021 Murray et al. https://creativecommons.org/licenses/by/4.0/ This content is distributed under the terms of the Creative Commons Attribution 4.0 International license.

10.1128/mSphere.00759-21.6 TABLE S2 Assembly 1 antiSMASH results for the 102 contigs of >40 kbp. The 72.1 and 78.1 region-encoded biosynthetic gene clusters presented two putative clusters that were further investigated using quantitative PCR (QPCR) to target samples with high BGC copy numbers for a subsequent round of metagenome sequencing. Download Table S2, PDF file, 0.02 MB. Copyright © 2021 Murray et al. 2021 Murray et al. https://creativecommons.org/licenses/by/4.0/ This content is distributed under the terms of the Creative Commons Attribution 4.0 International license.

We predicted several functional characteristics of the BGC that would be required for palmerolide A biosynthesis which aided our analysis (see reference 40) for detailed retrobiosynthetic analysis). This included evidence of a hybrid nonribosomal peptide-polyketide pathway and enzymatic domains leading to placement of two distinct structural features of the polyketide backbone, a carbamoyl transferase that appends a carbamate group at C-11 of the macrolide ring, and a hydroxymethylglutaryl-coenzyme A (CoA) synthase (HMGCoA) synthetase that inserts a methyl group on an acetate C-1 position of the macrolide structure (C-25). The antiSMASH results indicated that two of the three predicted hybrid NRPS *trans*-AT-type I PKS contained the predicted markers. Manual alignment of these two contigs suggested near-identical overlapping sequence (36,638 bases), and when joined, the merged contig resulted in a 74,672 kbp BGC (Fig. 1C). The cluster size was in the range of other large *trans*-AT PKS encoding BGCs, including pederin (54 kbp [41]), leinamycin (135.6 kbp [42]), as well as a *cis*-acting AT-PKS, jamaicamide (64.9 kbp [43]). The combined contigs encompassed what appeared to be a complete BGC that was flanked at the start with a transposase and otherwise unlinked in the assembly to other contiguous DNA. The cluster lacked phylogenetically informative marker genes from which putative taxonomic assignment could be attributed.

The antiSMASH results suggested that the BGC appears to be novel with the highest degree of relatedness to pyxipyrrolone A and B (encoded in the *Pyxidicoccus* sp. strain MCy9557 genome [44]), to which only 14% of the genes have a significant BLAST hit to genes in the metagenome-encoded cluster. The ketosynthase (KS) sequences (13 in all) fell into three different sequence groups (40). One was nearly identical (99% amino acid identity) to a previously reported sequence from a targeted KS study of *S. adareanum* microbiome metagenomic DNA (26). The other two were most homologous to KS sequences from Allochromatium humboldtianum and Dickeya dianthicola in addition to a number of hypothetical proteins from environmental sequence data sets. The primary polyketide synthase (PKS) component of the BGC (labeled contig 9 in Fig. 1C) includes 11 of the 13 KS domains, which is consistent with a polyketide backbone which has 22 contiguous carbons since each elongation step adds a two-carbon unit. This further supports the hypothesis that this candidate BGC is likely responsible for palmerolide A biosynthesis. A detailed bioinformatic analysis into the stepwise biosynthetic mechanism was conducted by Avalon et al. (40).

### Taxonomic inference of palmerolide A BGC.

**FIG 2:**
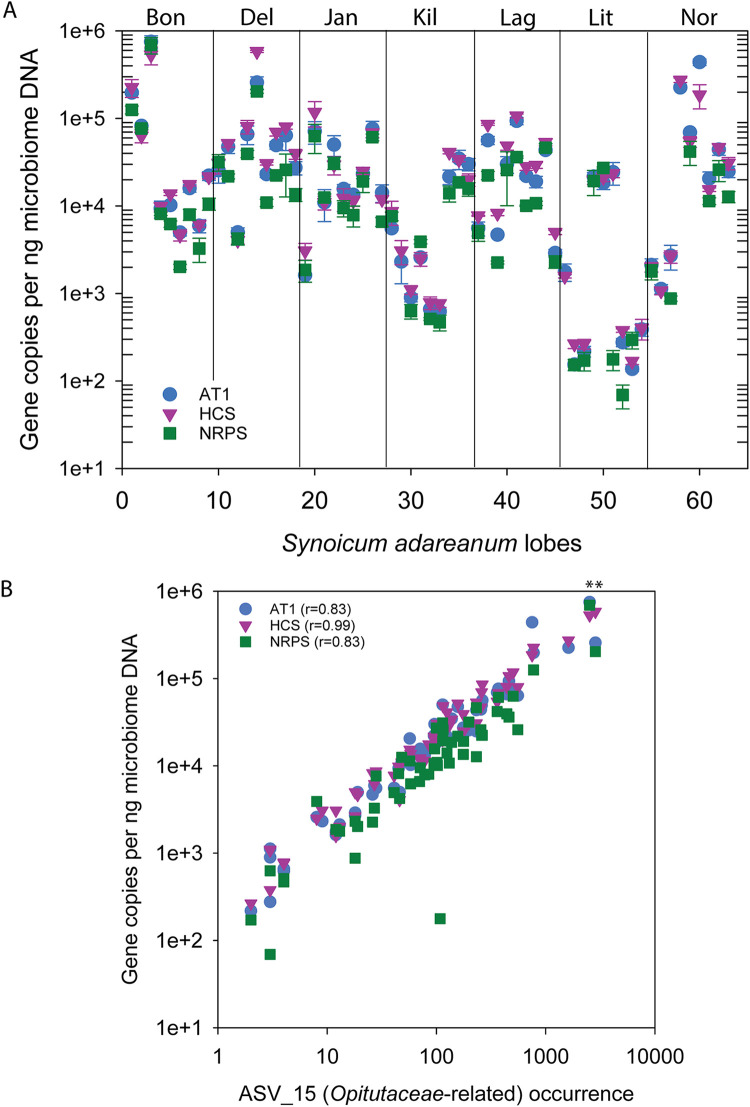
Abundances of real-time PCR-targeted coding regions in the candidate *pal* biosynthetic gene cluster in Antarctic ascidian samples. (A) Gene copies estimated for three targeted coding regions (acyltransferase AT1, 3-hydroxy-methyl-glutaryl coenzyme A synthase [HCS], and the condensation domain of a nonribosomal peptide synthase [NRPS]) in the candidate *pal* biosynthetic gene cluster surveyed over 63 DNA extracts derived from microbial cell preparations enriched from the Antarctic ascidian *Synoicum adarenum.* Nine samples were collected at each of seven sites: Bonaparte Point (Bon), Delaca Island (Del), Janus Island (Jan), Killer Whale Rocks (Kil), Laggard Island (Lag), Litchfield Island (Lit), and Norsel Point (Nor) (27). (B) Relationship between gene copy number for the three gene targets and the 16S rRNA gene ASV occurrences of *Opitutaceae*-related ASV_15 across a 63 *S. adareanum* microbial DNA sample set. Asterisks indicate samples Bon-1C-2011 and Del-2b-2011 that were selected for PacBio sequencing.

Taxonomic attribution of the BGC was inferred using a real-time PCR strategy targeting three coding regions of the putative palmerolide A BGC spanning the length of the cluster (acyltransferase AT1, hydroxymethylglutaryl Co-A synthase [HCS], and the condensation domain of the nonribosomal peptide synthase NRPS; Fig. 1C) to assay a *Synoicum* microbiome collection of 63 samples that have been taxonomically classified using Illumina small-subunit (SSU) rRNA gene tag sequencing (27). The three gene targets were present in all samples ranging within and between sites at levels from ∼7 × 10^1^ to 8 × 10^5^ copies per gram of host tissue (Fig. 2A). The three BGC gene targets covaried across all samples (*r*^2^ > 0.7 for all pairs), with the NRPS gene copy levels slightly lower overall (mean, 0.66 and 0.59 copies per ng host tissue for NRPS:AT1 and NRPS:HCS, respectively, *n* = 63). We investigated the relationship between BGC gene copies per nanogram of host tissue for each sample and palmerolide A levels determined for the same samples using mass spectrometry; however, no correlation was found (*R* < 0.03 and *n* = 63 [27]). We then assessed the semiquantitative relationship between the occurrence of SSU rRNA amplicon sequence variants (ASVs) (*n* = 461) (27) and the abundance of the three palmerolide A BGC gene targets. Here, we found a robust correlation (*R* = 0.83 to 0.99) between all three gene targets and a single amplicon sequence variant (ASV15) in the core microbiome (45). This ASV is affiliated with the *Opitutaceae* family of the *Verrucomicrobium* phylum. The *Opitutaceae* family ASV (SaM_ASV15) was a member of the core microbiome, as it was detected in 59 of the 63 samples surveyed at various levels of relative abundance and displayed strong correlations with the abundances of BGC gene targets (Fig. 2B, *r*^2^ = 0.68 with AT1, 0.97 with HCS, and 0.69 with NRPS, *n* = 63 for all). The only other correlations *R* > 0.5 were ASVs associated within the “variable” fraction of the microbiome, e.g., one low-abundance ASV was present in 24 of 63 samples (45).

This result supports the finding of Murray et al. (27) in which gene abundance and natural product chemistry do not reflect a 1:1 ratio in this host-associated system. Neither the semiquantitative measure of ASV copies nor the real-time PCR abundance estimates of the three biosynthetic gene targets correlated with the mass-normalized levels of palmerolide A present in the same samples. As discussed (27), this is likely a result of bioaccumulation in the ascidian tissues. This result provided strong support that the genetic capacity for palmerolide A production was associated with a novel member of the *Opitutaceae*, a taxonomic family with representatives found across diverse host-associated and free-living ecosystems. Although the biosynthetic capacity of this family is not well-known (46), recent evidence (36) suggests that this family may be a fruitful target for cultivation efforts and natural product surveys.

### Assembly of the palmerolide BGC-associated *Opitutaceae*-related metagenome-assembled genome (MAG).

With metagenomes, some genomes come together easily—while others present compelling puzzles to solve. Assembly of the *pal* BGC-containing *Opitutaceae* genome was the result of a dedicated effort of binning contigs, gene searches, additional sequencing of samples with high BGC titer, and manual, targeted assembly. Binning efforts with CoAssembly 1 did not result in association of the *pal* BGC with an associated metagenome-assembled genome (see Text S1 and Table S3 in the supplemental material). Therefore, a further round of metagenome sequencing using long-read technology (Pacific Biosciences Sequel Systems technology; PacBio) ensued.

10.1128/mSphere.00759-21.1 TEXT S1 Supplemental Materials and Methods. Details concerning ascidian sample collections, sample processing and high-molecular-weight DNA extraction, metagenome sequencing, metagenome binning, bin taxonomic and functional classification, real-time PCR, manual assembly procedures, annotation and phylogenomic analyses, and associated references are provided. Download Text S1, PDF file, 0.1 MB. Copyright © 2021 Murray et al. 2021 Murray et al. https://creativecommons.org/licenses/by/4.0/ This content is distributed under the terms of the Creative Commons Attribution 4.0 International license.

10.1128/mSphere.00759-21.7 TABLE S3 Quality checks and taxonomic classification using CheckM and GTDB-Tk for *Opitutaceae*-associated bins at different stages of analysis (most recent, highest quality on the left-most column and initial Coassembly 1 and bin 4 in the right-most column). The *Ca*. Synoicihabitans palmerolidicus-2 column reflects identifications (MetaERG annotation) of many of the missing markers in *Ca*. Synoicihabitans palmerolidicus-1. The *Opitutaceae* bin 8 – clean CoAssembly 2 data set reflects an effort to remove non-*Opitutaceae* sequences from the bin (see Text S1 in the supplemental material). CoAssembly 1 bins 1 and 2 included the palmerolide A biosynthetic gene cluster and were otherwise populated by short contigs and were taxonomically unidentified. CoAssembly 1 bin 4 included *Opitutales*-related contigs, including a SSU rRNA gene sequence. Download Table S3, PDF file, 0.03 MB. Copyright © 2021 Murray et al. 2021 Murray et al. https://creativecommons.org/licenses/by/4.0/ This content is distributed under the terms of the Creative Commons Attribution 4.0 International license.

The 16S rRNA gene ASV occurrence (27) and real-time PCR data were used to guide *S. adareanum* sample selection for sequencing. Two ascidian samples (Bon-1C-2011 and Del-2B-2011) with high *Opitutaceae* ASV occurrences (ASV_015; >1,000 sequences each—relative abundance of ∼13.3% to 15.3% compared to an overall average of 1.3% ± 2.77% across the 63 samples, respectively) (27) and high BGC gene target levels (i.e., Bon-1C-2011 (mean number of copies per nanogram ± standard deviation [SD], *n* = 3): (6.93 ± 1.48) × 10^5^ NRPS; (7.52 ± 1.29) × 10^5^ AT1; (5.32 ± 1.22) × 10^5^ HCS and Del 2B-2011 (copies per ng ± SD, *n* = 3): (2.04 ± 0.10) × 10^5^ NRPS; (2.57 ± 0.41) × 10^5^ AT1; (5.84 ± 0.16) × 10^5^ HCS) were selected for PacBio sequencing. This effort generated 28 GB of data that was used to create a new hybrid CoAssembly 2 which combined all three sequencing technologies. Similar to the assembly with the Mycale hentscheli-associated polyketide producers (47), the long-read data set improved the assembly metrics, and subsequent binning resulted in a highly resolved *Opitutaceae*-classified bin (45) (Table S3). Interestingly, however, the palmerolide BGC contigs still did not cluster with this bin, which we later attributed to binning reliance on sequence depth.

**FIG 3:**
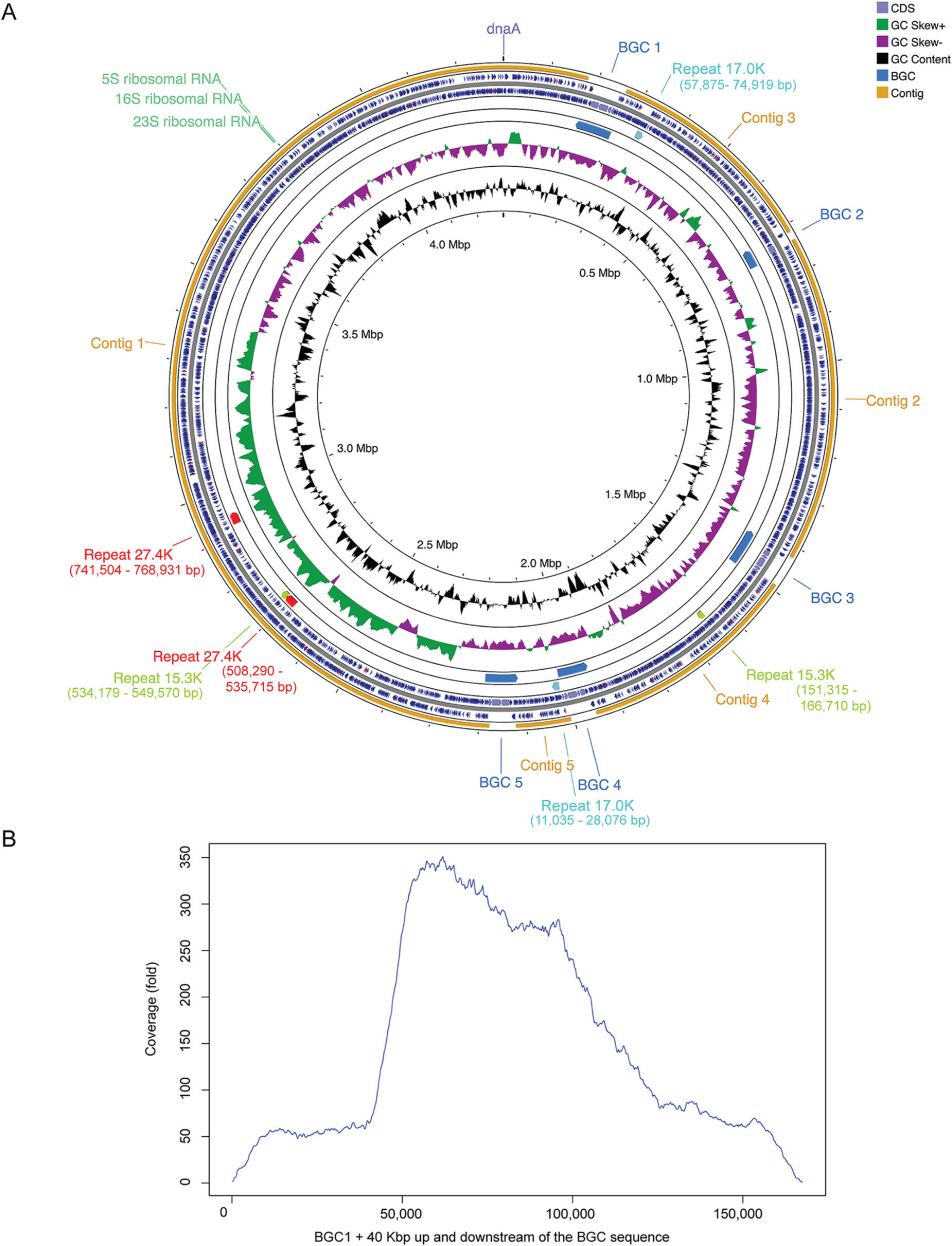
Genome maps of assembled MAG, *Candidatus* Synoicohabitans palmerolidicus and evidence of multicopy biosynthetic gene clusters. (A) The 4,297,084-bp gene map is oriented to *dnaA* at the origin. One possible assembly scenario of the *Ca.* Synoicihabitans palmerolidicus genome is shown as the order of the contigs and palmerolide BGCs are not currently known. In addition to the five BGCs, three other internally repetitive regions were identified (15.3, 17.0, and 27.4 kbp). The genes and orientation are shown in blue, and tRNAs are indicated in red. (B) To demonstrate the depth of coverage outside and inside the BGC regions, CCS reads from the Bon-1C-2011 and Del-2b-2011 samples were mapped to a 167.6-kbp region. The profile extends 40 kbp into the genome on either side of the BGC where depth of coverage averages 60-fold, while in the BGC, depth of coverage varies across the BGC, given differences in cover across the BGC. The highest cover is 5×, or ∼300-fold, supporting the finding of five repeats encoding the BGC.

We used PacBio circular consensus sequence (CCS) reads to generate and manually edit the assembly for our *Opitutaceae* genome of interest. The resulting 4.3-Mbp genome (Fig. 3A) had a GC content of 58.7% and was resolved into a total of 10 contigs. Five of the contigs (contigs 1 to 5) were unique, while contigs 6 to 10 represented highly similar repeated units of the *pal* BGC (labeled *pal* BGC 1, 2, 3, 4, and 5) beginning and ending in linkage gaps. Support for the assembly of a nearly complete genome includes manual analysis of the ends of the five unique contigs and underlying read data linking each end of all five contigs to parts of the repeated contigs. The structures of the five repeated contigs were manually evaluated (Fig. 1), and while each copy had unique features, all were found to harbor portions of the palmerolide BGC. While the exact placement of each of the five palmerolide contigs could not be positioned with respect to the five unique contigs, the data support a single circular chromosome. Analyses further supporting a complete genome include the presence of a complete ribosomal operon and estimated CheckM completeness of 96.04% based on marker genes (the presence of marker genes were identified using MetaERG annotation of the *Opitutaceae* genome; Table S3) and 45 tRNA genes.

Alignment of all five repeated contigs to the longest palmerolide-containing BGC revealed a long (36,198-base) repeated region that was shared between all five contigs with some substantial differences at the beginning of the cluster and only minor differences at the end, indicating three full-length and two shorter palmerolide BGC-containing contigs (Fig. 1 and 3). This was consistent with coverage estimates based on read mapping that suggested lower depth at the beginning of the cluster (Fig. 3B). BGC 1 and 3 are nearly identical (over 86,135 bases) with only two single nucleotide polymorphisms (SNPs) and an additional 1,468 bases in BGC 1 (237 bases at the 5′ end and 1,231 bases at the 3′ end). BGC 4 is 13,470 bases shorter than BGC 1 at the 5′ end and 5 bases longer than BGC 1 at the 3′ end. Alignment of the real-time PCR gene targets to the five *pal* BGCs provided independent support for the different lengths of the five BGCs, as the region targeted by the NRP primers was missing in two of the *pal* BGCs, thus explaining lower NRP:AT or NRP:HCS gene dosages reported above.

Interestingly, precedent for naturally occurring multicopy BGCs to our knowledge has only recently been found in one other bacterium, an ascidian (*Lissoclinum* sp.)-associated *Opitutaceae*, *Candidatus* Didemnitutus mandela, linked to cytotoxic mandelalide polyketides (36). Likewise, we can invoke a rationale similar to the rationale in reference 36 that multiple gene clusters may be linked to biosynthesis of different palmerolide derivatives; see Avalon et al. (40) for retrobiosynthetic predictions of these clusters and annotation of putative enzymatic functions. Gene duplication, loss, and rearrangement processes over evolutionary time likely explain the source of the multiple copies. At present, we do not yet understand the regulatory controls, whether all five are actively transcribed, *in situ* function, and how this may vary among host microbiomes.

### Phylogenomic characterization of the *Opitutaceae*-related MAG.

The taxonomic relationship of the *Opitutacae* MAG to other *Verrucomicrobia* was assessed using distance-based analyses with 16S rRNA gene and average amino acid identity (AAI). Then, it was classified using the GTDB-Tk tool (48) and a phylogenomic analysis based on concatenated ribosomal protein markers. Comparison of 16S rRNA gene sequences among other *Verrucomicrobia* with available genome sequences (that also have 16S rRNA genes; Fig. S1) suggests that the nearest relatives are Cephaloticoccus primus CAG34 (similarity of 0.9138), Optitutus terrae PB90-1 (similarity of 0.9132), and Geminisphaera coliterminitum TAV2 (similarity of 0.9108). The *Opitutaceae*-affiliated MAG sequence is identical to a sequence (uncultured bacterium clone Tun-3b A3) reported from the same host (*S. adareanum*) in a 2008 study (26); bootstrapping supported a deep branching position in the *Opitutaceae* family. Thus, this *Opitutaceae*-related MAG appears to be unique—its 16S rRNA gene sequence was not found in bacterioplankton amplicon surveys from the Anvers Island archipelago (*n* = 604 amplicon sequences) and a culture collection reported previously (27), nor were any sequences with identities higher than 95% found following against a large bacterioplankton amplicon data set from further north in the Antarctic Peninsula (32,941 sequence clusters derived from 44.6 million sequences; NCBI Bioproject accession no. PRJNA316748). Likewise, when searched against the global nr GenBank database representing environmental data sets, the highest sequence identities found so far are <96% identity over 88% of the complete sequence.

10.1128/mSphere.00759-21.2 FIG S1 rRNA-based phylogenetic tree. Sequences were selected from *Verrucomicrobia* (phylum) and mostly *Opitutaceae* (family) genomes represented by isolates, metagenome-assembled genomes and single-cell-assembled genomes from host-associated and marine ecosystems. The RAXML tree is based on 1,636 bases; 250 bootstraps were run, and values of >49 are shown at nodes. *Ca*. Synoicihabitans palmerolidicus (*Opitutaceae* bin 8) is indicated with an orange diamond symbol. Symbols designate environmental origins of the organisms: free-living organisms are represented by circles, and the sources of the organisms are indicated as follows: light blue, marine; green, freshwater; red, hydrothermal mud; brown, soils. Host-associated taxa from marine systems (blue diamonds) and from terrestrial systems (black diamonds) are indicated. Download FIG S1, PDF file, 0.3 MB. Copyright © 2021 Murray et al. 2021 Murray et al. https://creativecommons.org/licenses/by/4.0/ This content is distributed under the terms of the Creative Commons Attribution 4.0 International license.

When characterizing the MAG using AAI metrics (average nucleotide identity [ANI] found no closely related genomes), the closest genomes were environmental metagenome assemblies from the South Atlantic TOBG_SAT_155 (53.08% AAI) and WB6_3A_236 (52.71% AAI), and the two closest isolate type genomes were Nibricoccus aquaticus strain NZ CP023344 (52.82% AAI) and Opitutus terrae strain PB90 (52.75% AAI). The Microbial Genome Atlas (MiGA) support for the MAG belonging in the *Opitutaceae* family was weak (*P* values of 0.5). Attempts to classify this MAG using GTDB-Tk (47) were hampered by the fact we have no real representative in the genome databases, resulting in low-confidence predictions at the species or genus level (see the supplemental material for details).

**FIG 4:**
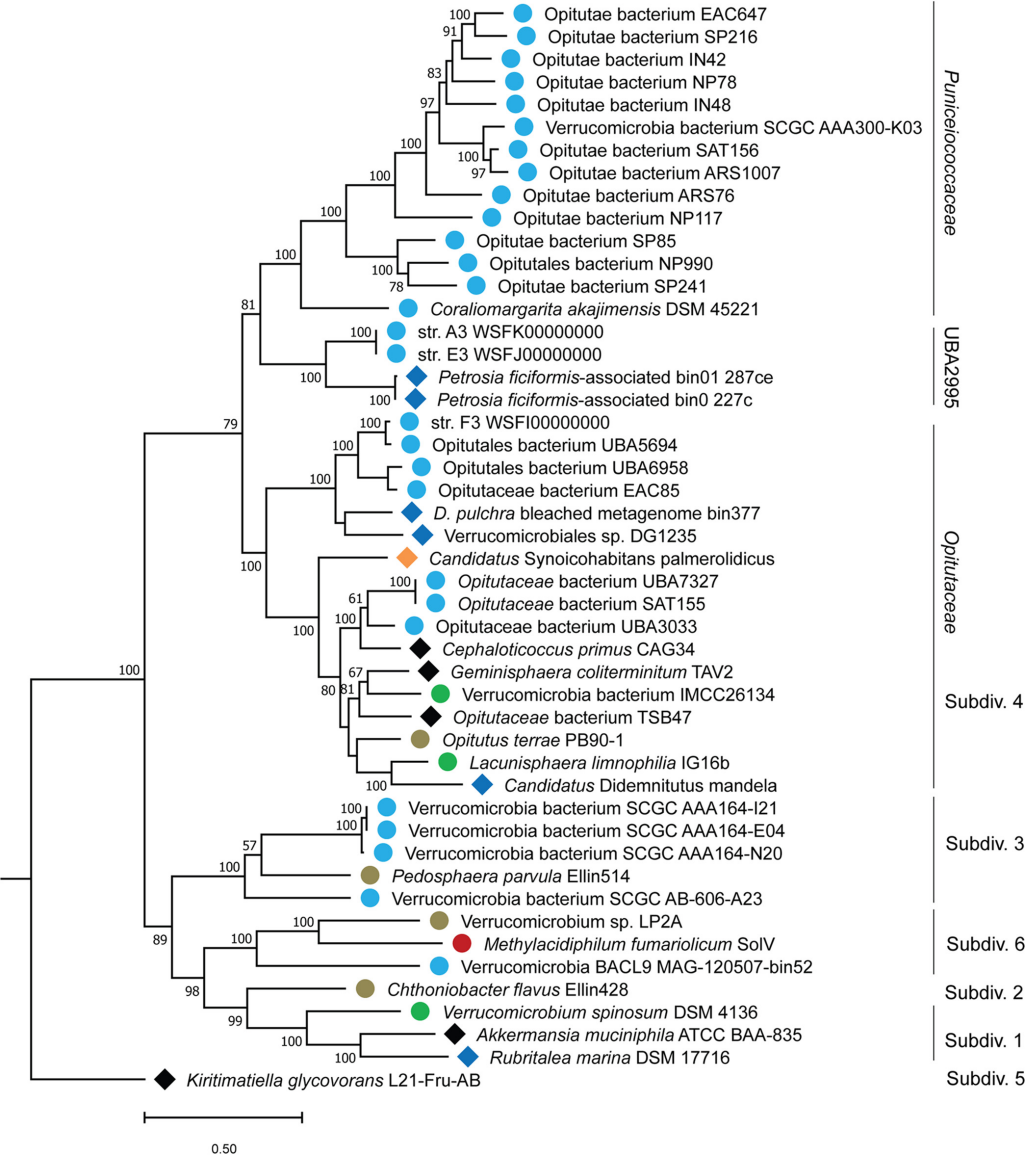
Maximum likelihood phylogenomic tree showing 48 *Verrucomicrobia* genomes. Phylogenomic relationship of *Candidatus* Synoicohabitans palmerolidicus (*Opitutaceae* bin 8) with respect to other mostly marine, and host-associated *Verrrucomicrobia* subdivision 4 and other genomes. The tree is based on 16 concatenated ribosomal proteins (5,325 amino acids) common across 48 *Verrucomicrobia* genomes. Distance was estimated with RAxML with 300 bootstrap replicates. Symbols designate environmental origins of the organisms. Free-living organisms are represented by circles. Marine (light blue), freshwater (green), hydrothermal mud (red), and soil (brown) organisms are indicated. Host-associated taxa from marine systems (blue diamonds) and from terrestrial systems (black diamonds) are indicated. Subdiv., subdivision.

Verrucomicrobia exhibit free-living and host-associated lifestyles in a multitude of terrestrial and marine habitats on Earth. We performed a meta-analysis of *Verrucomicrobia* genomes, with an emphasis on marine and host-associated *Opitutaceae*, to establish more confidence in the phylogenetic position of the *Opitutaceae* MAG. The analysis was based on 24 conserved proteins—21 ribosomal proteins and 3 additional conserved proteins (InfB, LepA, and PheS). The diversity of the *Opitutaceae* family, and of *Verrucomicrobia* in general, is largely known from uncultivated organisms in which there are 20 genera in GTDB (release 05-RS95), 2 additional genera in the NCBI taxonomy database, and numerous unclassified single amplified genomes (SAGs); in all, only 8 genera have cultivated representatives. Given the uneven representations of the 24 proteins across all (115) genomes assessed (MAGs and SAGs are often incomplete), we selected a balance of 16 proteins across 48 genomes to assess phylogenomic relatedness across the *Opitutaceae* (Fig. 4). Here too, as seen with the 16S rRNA gene phylogenetic tree, the *S. adareanum*-*Opitutaceae* MAG held a basal position compared to the other *Opitutaceae* genomes in the analysis.

### *Ca.* Synoicihabitans palmerolidicus relative abundance estimates and ecological inference.

The relative abundance of *Opitutaceae* bin 8 was estimated in the shotgun metagenomic samples by mapping the NGS reads back to the assembled MAG across the four *S. adareanum* samples collected. This indicated various levels of genome coverage in the natural samples, with the two samples selected based on real-time PCR-quantified high BGC copy number being clearly enriched in this strain (44.70% of reads mapped to Bon-1C-2011 and 36.78% to Del-2B-2011 [Table 1]). These levels are higher than estimates of relative abundance derived from the 16S rRNA gene amplicon surveys (estimated at 13.33 and 15.34%, respectively) for the same samples. This is likely a result of the single-copy nature of the ribosomal operon in *Opitutaceae* bin 8 versus other taxa with multiple rRNA operon copies that could thus be overrepresented in the core microbiome library (e.g., *Pseudovibrio* sp. strain PSC04-5.I4 [NCBI WGS assembly FNLB01000000] has 9 and *Microbulbifer* sp. is estimated at 4.1 ± 0.8 based on 9 finished *Microbulbifer* genomes available at the Integrated Microbial Genomes Database). All host *S. adareanum* lobes surveyed (*n* = 63) in the Anvers Island regional survey contained high levels (0.49 to 4.06 mg palmerolide A × g^−1^ host dry weight) of palmerolide A (27), and variable, yet highly concordant levels of the *pal* BGCs and 16S rRNA ASV levels (Fig. 2). Despite the natural population structure sampled here (four single host lobes), the bin-level sequence variation was low (ranging from 72 to 243 SNPs) when the PacBio reads were mapped back to the *Opitutaceae* bin 8 (Table 1). This suggests maintenance of a relatively invariant population at the spatial and temporal scale of this coastal Antarctic region while highlighting our limited understanding of the biogeographical extent of the *S. adareanum*-symbiont-palmerolide relationship across a larger region of the Southern Ocean.

**TABLE 1:** Metagenomic reads from four different samples were mapped back to the *Ca.* Synoicihabitans palmerolidicus MAG

Ca. Synoicum adareanum sample	Technology	No. of reads	Mapped reads		Base coverage	Avg fold	No. of gaps	No. of gap bases	No. of SNPs	No. of indels
			No.	%						
Bon-1c-2011	PacBio CCS reads (1 cell)	48,298	21,591	44.70	99.98	58.38	2	644	72	126
Del-2b-2011	PacBio CCS reads (3 cells)	9,576	3,522	36.78	99.89	8.43	3	4,618	196	64
Nor-2c-2007	454	1,570,126	23,993	1.53	90.15	2.79	1,734	422,870	168	17
Nor-2a-2007	Ion Torrent Proton	89,330,870	15,979,084	17.89	99.98	708.79	8	774	243	68

Several questions remain with regard to the *in situ* function of palmerolide A (a eukaryotic V-ATPase inhibitor in human cell line assays [28]) in this cryohabitat: how and why is it bioaccumulated by the host? Overall, the study of natural products in high-latitude marine ecosystems is in its infancy. This palmerolide-producing, ascidian-associated, *Opitutaceae* provides the first Antarctic example in which a well-characterized natural product has been linked to the genetic information responsible for its biosynthesis. Gaining an understanding of environmental and biosynthetic regulatory controls, establishing integrated transcriptomic, proteomic, and secondary metabolome expression in the environment will also reveal whether the different clusters are expressed *in situ*. In addition to ecological pursuits, the path to clinical studies of palmerolide will require genetic or cultivation efforts. At present, we hypothesize that cultivation of *Opitutaceae* bin 8 may be possible, given the lack of genome reduction or of other direct evidence for host-associated dependencies (e.g., a number of central carbohydrate and energy metabolism pathways appear to be present).

### *Candidatus* Synoicihabitans palmerolidicus genome attributes.

The Antarctic ascidian, *Synoicum adareanum*, harbors a dense community of bacteria that has a conserved core set of taxa (27). The near-complete ∼4.30-Mbp *Opitutaceae* bin 8 metagenome-assembled genome (Fig. 3) represents one of the core members. This MAG is remarkable in that it encodes five 36- to 74-kbp copies of the candidate BGCs that are implicated in biosynthesis of palmerolide A and possibly other palmerolide compounds. Intriguingly, this genome does not seem to show evidence of genome reduction as found in *Candidatus* Didemnitutus mandela (36); the other ascidian-associated *Opitutaceae* genome currently known to encode multiple BGC gene copies. This is the first *Opitutaceae* genome characterized from a permanently cold, ca. −1.8 to 2°C, often ice-covered ocean ecosystem. This genome encodes one rRNA operon, 45 tRNA genes, and an estimated 5,058 coding sequences. Based on the low (<92%) SSU rRNA gene identity and low (<54% AAI) values to other genera in the *Opitutaceae*, along with the phylogenomic position of the *Opitutaceae* bin 8, the provisional name “*Candidatus* Synoicihabitans palmerolidicus” (*Ca*. Synoicihabitans palmerolidicus) is proposed for this novel verrucomicrobium. The genus name *Synoicihabitans* (Syn.o.i.ci.ha’bitans. N.L. neut. N. *Synoicum* a genus of ascidians; L. pres. part *habitans* inhabiting; N.L. masc. n.) references this organism as an inhabitant of the ascidian genus *Synoicum*. The species name *palmerolidicus* (pal.me.ro.li’di.cus. N.L. neut. n. *palmerolidum* palmerolide; N.L. masc. adj.) designates the species as pertaining to palmerolide.

The GC content of 58.7% is rather high compared to other marine *Opitutaceae* genomes (average, 51.49; SD, 0.02; *n* = 12), yet is approximately average for the family overall (61.58; SD, 0.06; *n* = 69; Table S4). MetaERG includes metagenome-assembled genomes available in the GTDB as a resource for its custom GenomeDB that new genomes are annotated against. This was a clear advantage in annotating the *Ca.* Synoicihabitans palmerolidicus genome as *Verrucomicrobia* genomes are widely represented by uncultivated taxa. Likewise, antiSMASH was an invaluable tool for *pal* BGC identification and domain structure annotation. This formed the basis to derive a predicted stepwise mechanism of *pal* biosynthesis (40).

10.1128/mSphere.00759-21.8 TABLE S4 GC percent for *Opitutaceae* family representatives. Different genera are represented with different colors. Marine *Optitutaceae* metagenome-assembled genomes are indicated with an asterisk. Source and species nomenclature are from GTDB release 05-RS95. The GC content for *Ca.* Synoicihabitans palmerolidicus is 58.7%. Download Table S4, PDF file, 0.04 MB. Copyright © 2021 Murray et al. 2021 Murray et al. https://creativecommons.org/licenses/by/4.0/ This content is distributed under the terms of the Creative Commons Attribution 4.0 International license.

### *Ca*. Synoicihabitans palmerolidicus genome structure, function, and host-associated features.

Beyond the *pal* BGCs, the *Ca*. Synoicihabitans palmerolidicus genome encodes a variety of additional interesting structural and functional features that provide insight into its lifestyle. Here, we provide a brief synopsis. In addition to the repeated BGCs, three additional repeated elements with two nearly identical copies each (15.3 kbp, 17.0 kbp, and 27.4 kbp) were identified during the assembly process (Fig. 3A). These elements coded for 20, 25, and 41 coding sequences (CDSs), respectively, were in some cases flanked by transposase/integrases (both internal and proximal), and had widespread homology with *Verrucomicrobia* orthologs. The contents of the three repetitive elements were not shared among each other.

Annotations were assigned to a little more than half of the CDSs in the 15.3-Mbp repeat which is predicted to encode for xylose transport, two sulfatases, two endonucleases, and a MacB-like (potential macrolide export) periplasmic core protein. Xylan might be sourced from seaweeds (49) or even the ascidian, as it is a minor component of the tunic cellulose (50). Related to this, an endo-1,4-beta-xylanase which has exoenzyme activity in some microorganisms (51) was identified elsewhere in the genome, suggesting the potential for xylose metabolism. Altogether, eight sulfatase copies were identified in this host-associated genome (four in the 15.5-Mbp repeat elements). These may be involved in catabolic activities of sulfonated polysaccharides, and possibly as *trans*-acting elements in palmerolide biosynthesis (40). In addition to the MacB-like CDSs found in this repeat, 13 other MacB homologs were present in the genome—none of which were associated with the *pal* BGCs (Fig. S2a). MacB is a primary component of the macrolide tripartite efflux pump that operates as a mechanotransmission system involved both in antibiotic resistance and antibiotic export depending on the size of the macrolide molecule (52). However, two additional elements required for this pump to be functional, an intramembrane MacA and an outer membrane protein TolC, were not colocated elsewhere in the genome. MacA may be missing, as searches against the *Ca*. Synoicihabitans palmerolidicus genome with two other verrucomicrobia-associated MacA CDSs were not identified using BLAST queries (peat soil MAG SbV1 SBV1_730043 and *Ca*. Udaeobacter copiosis KAF5408997.1 [53]). At least nine MacB CDSs were flanked by a FstX-like permease family protein, the genomic structures of which were quite complex, including several with multiple repeated domains. Detailed transporter modeling is beyond the scope of this work, but it is likely that these proteins are involved in signaling of cell division machinery rather than macrolide transport (54).

10.1128/mSphere.00759-21.3 FIG S2 Phylogenetic relationships of homologs of (A) MacB CDS and (B) celluase glycosylhydrolase family 5 enzymes identified in the *Ca*. Synoicihabitans palmerolidicus genome and closest neighboring sequences that were sourced from GenBank using BLAST. Maximum likelihood analysis was conducted using RAxML v. 8.2.12 using the PROTGAMMALG model with 550 bootstrap replicate trees calculated for panel A and 1,000 bootstrap replicate trees calculated for panel B. Bootstrap values of >49 are shown which represent the percentage of times the topology was found. Download FIG S2, PDF file, 0.2 MB. Copyright © 2021 Murray et al. 2021 Murray et al. https://creativecommons.org/licenses/by/4.0/ This content is distributed under the terms of the Creative Commons Attribution 4.0 International license.

Predicted CDSs in the 17.0-Mbp repeat included sugar binding and transport domains, as well as domains encoding rhamnosidase, arabinofuranosidase, and other carbohydrate catabolism functions. About half the proteins encoded in the 27.4-Mbp repeat were unknown in function, and for the remaining characterized proteins, diverse potential functional capacities were suggested. For example, a zinc carboxypeptidase (one of three in the genome), multidrug and toxic compound transporter (MatE/Norm), and an exodeoxyribonuclease were identified.

The *Ca.* Synoicihabitans palmerolidicus genome has a number of features that suggest it is adapted to a host-associated lifestyle, and several of these features were reported recently for two related sponge-associated *Opitutales* metagenome bins (Petrosia ficiformis*-*associated bins 0 and 01, Fig. 4) (55). These include identification of a bacterial microcompartment (BMC) “super locus.” Such loci were recently reported to be enriched in host-associated *Opitutales* genomes compared to free-living relatives. The structural proteins for the BMC were present as were other conserved *Planctomyces*-*Verrucomicrobia* BMC genes (56). As in the sponge Pectoria ficiformis metagenome bins, enzymes for carbohydrate (rhamnose) catabolism and modification were found adjacent to the BMC locus (Fig. S3), in addition to the two that were found in the 27.4-Mbp repeat. The genome did not appear to encode the full complement of enzymes required for fucose metabolism, though a few alpha-l-fucosidases were identified. Further evidence for carbohydrate metabolism was supported through genome similarity searches with the CAZy database (57), including 7 identified carbohydrate binding modules, a carbohydrate esterase, 14 glycoside hydrolases, 6 glycosyl transferases, and a polysaccharide lyase. In addition, three bacterial cellulases (PF00150, cellulase family A; glycosyl hydrolase family 5) were identified, all with the canonical conserved glutamic acid residue. These appear to have different evolutionary histories in which each variant has nearest neighbors in different bacterial phyla (Fig. S2b) matching between 68% identity for protein J6386 03765 to Lacunisphaera limnophila, 57.5% identity for protein J6386 22340 with a cellulase from a shipworm symbiont *Alteromonadaceae* (*Terridinibacter* sp.), and 37.5% sequence identity to a *Bacteroidetes* bacterium. This suggests the potential for cellulose degradation—which is consistent with ascidians being the only animals known to produce cellulose for its skeletal structure (50). In addition to the BGCs, the enzymatic resources in this genome (e.g., xylan and cellulose hydrolysis) are a treasure trove rich with biotechnological potential. Support for a type II secretion system (i.e., GspD, GspE, GspG, and GspO), common to Gram-negative bacteria which secrete folded proteins (e.g., hydrolytic enzymes required for survival in host environments) from the periplasm into the extracellular environment (58) were detected in the *Ca*. Synoicihabitans palmerolidicus genome.

10.1128/mSphere.00759-21.4 FIG S3 Bacterial microcompartment (BMC) superloci identified in the *Ca.* Synoicihabitans palmerolidicus MAG. Conserved structural elements identified with Pfam and Swiss-Prot annotations were consistent with other *Planctomycete-Verrucomicrobia* BMC (PV-BMC) loci which are associated carbohydrate utilization domains. Transcriptional regulatory genes are indicated in green, conserved PV-BMC enzymes in blue, the BMC proteins in red, enzymes associated with rhamnose metabolism in violet, and those with lactate utilization in purple. Download FIG S3, PDF file, 0.02 MB. Copyright © 2021 Murray et al. 2021 Murray et al. https://creativecommons.org/licenses/by/4.0/ This content is distributed under the terms of the Creative Commons Attribution 4.0 International license.

Chemotaxis and flagellar biosynthesis are factors required for horizontally acquired symbionts, as has been established with the Vibrio fischeri*-*Euprymna scolopes symbiosis (59). The *Ca*. Synoicihabitans palmerolidicus genome encodes a chemotaxis system (e.g., CheA, CheW, CheR, CheB, CheC, and methyl-accepting chemotaxis domains) with flagellar motors in addition to a number of other elements of flagellar biosynthesis, which is consistent with horizontal acquisition by the host. The methyl-accepting chemotaxis system in V. fischeri responds to chitobiose production by E. scolopes (59)—it will be interesting to further unravel the details of *Ca*. Synoicihabitans palmerolidicus cellular biology to understand the chemotaxis stimulants, preferential cellular localization of palmerolide production and resistance mechanisms of the host to the potent vacuolar ATPase, as well as products made by others in the *S. adareanum* microbiome.

Other indicators of host association and palmerolide production include T-A domains, multidrug exporters, and the potential for palmerolide transport and cofactor biosynthesis. T-A domains were also prevalent in the *Petrosia ficiformis-*associated bins 0 and 01 (55). The *Ca*. Synoicihabitans palmerolidicus genome encoded at least 22 TA-related genes, including multiple MazG and AbiEii toxin type IV TA systems, AbiEii-Phd_YefM type II toxin-antitoxin systems, along with genes coding for PIN domains, Zeta toxin, RelB, HipA, MazE, and MraZ. In addition to the MatE (found in the 27.4-Mbp repeat), two other multidrug export systems with homology to MexB and MdtB were identified. This analysis also resulted in identifying a putative AbiEii toxin (PF13304) with homology to SyrD, a cyclic peptide ABC type transporter that was present in all five BGCs (Fig. 1C; with 52.7% BLAST percent identity to a *Desulfamplus* sp. homolog over the full length of the protein, and a variety of other bacteria, including an *Opitutaceae*-related strain with similar levels of identity). This transporter is encoded downstream of the large polyketide gene clusters following the acyl transferase domains and precedes the predicted *trans*-acting domains at the 3′ end of the BGC. Given its genomic position, this protein is a candidate for palmerolide transport. The *Ca*. Synoicihabitans palmerolidicus genome also encodes the potential for pantothenate biosynthesis via *ilvD*, *ilvE*, *panB*, *ilvC*, *panD*, and *panC* (60), which is consistent with palmerolide biosynthesis in which the 4′-phosphopantetheinyl prosthetic group interacts with acyl carrier proteins for multimodular assembly. Some symbionts, e.g., *Ca*. Entotheonella sp., are auxotrophic for pantothenate and likely acquire it from other microbiome members (61).

Unlike *Ca*. Didemnitutus mandela (36), there does not appear to be ongoing genome reduction, which may suggest that the *S. adareanum*-*Ca*. Synoicihabitans palmerolidicus relationship is more recent, and/or that the relationship is commensal rather than interdependent. Likewise, we suspect that the pseudogene content may be high, as several CDSs appear to be truncated, in which redundant CDSs of various lengths were found in several cases (including MacB). There is evidence of lateral gene transfer acquisitions of cellulase and numerous other enzymes that may confer ecological advantages through the evolution of this genome. Similarly, the origin of the *pal* BGCs and how recombination events play out in the success of this Antarctic host-associated system in terms of adaptive evolution (62), not to mention the ecology of *S. adareanum*, are curiosities. This phylum promises to be an interesting target for further culture-based and cultivation-free studies—particularly in the marine environment, in which they have been less well studied compared to terrestrial free-living and host-associated systems.

Together, it appears that the genome of *Ca*. Synoicihabitans palmerolidicus is equipped for life in this host-associated interactive ecosystem that stands to be one of the first high-latitude marine invertebrate-associated microbiomes with a genome-level understanding—and one that produces a highly potent natural product, palmerolide A. This system holds promise for future research now that we have identified the palmerolide A-producing organism and *pal* BGC. We still have much to learn about the ecological role of palmerolide A—if it is involved in predation avoidance, antifouling, antimicrobial defense, or some other yet to be recognized aspect of life in the frigid, often ice-covered, and seasonally light-limited waters of the Southern Ocean.

## MATERIALS AND METHODS

### Sample collection.

Synoicum adareanum lobes were collected in the coastal waters off Anvers Island, Antarctica, and stored at −80°C until processing (see Table S1 in the supplemental material). See Supplemental Text S1 and reference 27 for details of sample collection, microbial cell preparation, and DNA extraction.

### Metagenome sequencing.

Three rounds of metagenome sequencing were conducted, the details of which are in Text S1 in the supplemental material. This included an initial 454 pyrosequencing effort with a bacterium-enriched metagenomic DNA preparation from S. adareanum lobe (Nor2c-2007). Next, an Ion Proton System was used to sequence a metagenomic DNA sample prepared from *S. adareanum* lobe Nor2a-2007. Then two additional *S. adareanum* metagenome DNA samples (Bon-1C-2011 and Del-2b-2011) were selected based on high copy numbers of the palmerolide A BGC (see real-time PCR methods in Text S1 in the supplemental material) and sequenced using Pacific Biosciences Sequel Systems technology.

### Metagenome assembly, annotation, and binning.

Raw 454 metagenomic reads (1,570,137 single end reads, 904,455,285 bases) were assembled by Newbler (63) v2.9 (Life Technologies, Carlsbad, CA, flags: -large -rip -mi 98 -ml 80), while Ion Proton metagenomic reads (89,330,870 reads, 17,053,251,055 bases) were assembled using SPAdes (64) v3.5 (flags: --iontorrent). Both assembled data sets were merged with MeGAMerge (65) v1.2 and produced 86,387 contigs with a maximum contig size of 153,680 and total contig size of 144,953,904 bases (CoAssembly 1). To achieve more complete metagenome coverage and facilitate metagenome-assembled genome assembly, a circular consensus sequence (CCS) protocol (PacBio) was used to obtain high-quality long reads on two samples, Bon-1C-2011 and Del-2b-2011. The 5,514,426 PacBio reads were assembled with aforementioned assembled contigs (CoAssembly 1) on EDGE Bioinformatics using wtdbg2 (66), a fast and accurate long-read assembler. The contigs were polished with three rounds of polishing by Racon (67) into a second coassembly (CoAssembly 2) which has 4,215 contigs with a maximum contig size of 2,235,039 and total size of 97,970,181 bases. Last, a manual approach was implemented to arrive at assembly of the MAG of interest, the details of which are described in the supplemental material.

The contigs from both coassemblies 1 and 2 were submitted initially to the EDGE bioinformatics platform (68) for sequence annotation using Prokka (69) v1.13 and taxonomy classification using BWA (70) mapping to NCBI RefSeq (version: NCBI 3 October 2017). Bioinformatic predictions of natural product potential was performed using the antibiotics and secondary metabolite analysis shell (antiSMASH, bacterial versions 3.0, 4.0 and 5.0 (38, 39, 71). This tool executed contig identification, annotation, and analysis of secondary metabolite biosynthesis gene clusters on both CoAssemblies 1 and 2 (>1-kbp and >40-kbp data sets). As most of our attention was focused on analysis of the *Ca*. Synoicihabitans palmerolidicus assembled metagenome, we also used MetaERG (72) as the primary pipeline for metagenome annotation of the 10 final contigs in addition to NCBI’s PGAP pipeline (see reference 45) for the GFF data set. There were 5,186 coding sequences predicted in the MetaERG annotation and 5,186 in NCBI’s PGAP annotation.

MaxBin (73) and MaxBin2 (74) were used to form metagenome bins for both CoAssembly 1 and 2. CheckM v1.1.11 (75) and v.1.1.12 and GTDB-Tk v.1.0.2 (48) were used to verify bin quality and taxonomic classification. See supplemental material for details. In order to assess the representation of the assembled *Opitutaceae* genome across the four environmental samples used for metagenome sequencing (resulting from MaxBin2 binning of CoAssembly 2), we used BWA to map the CCS reads to each metagenome data set.

### Real-time PCR.

Gene targets (nonribosomal peptide synthase, acyltransferase, and 3-hydroxymethylglutaryl coenzyme A synthase) were selected at different positions along the length of the candidate BGC. Table S5 lists the primer and the GBlocks synthetic positive-control sequence. Metagenomic DNA extracts from a large *S. adareanum* sample set (*n* = 63 *S. adareanum* lobes from 21 colonies), all containing high levels of palmerolide A (27), were screened with the real-time PCR assays on a Quant Studio 3 (Thermo Fisher Scientific, Inc.; see Text S1 in the supplemental material for details of controls and analysis).

10.1128/mSphere.00759-21.9 TABLE S5 Quantitative PCR gene targets and primers. Primers targeting three gene targets were used in this study. Primer identifiers (IDs) are based on bases from the beginning of the CDS. ^†^A GBlocks synthetic DNA positive control was used for estimating copy number. Download Table S5, PDF file, 0.02 MB. Copyright © 2021 Murray et al. 2021 Murray et al. https://creativecommons.org/licenses/by/4.0/ This content is distributed under the terms of the Creative Commons Attribution 4.0 International license.

### Phylogenomic analyses.

A phylogenomic analysis of the assembled *Opitutaceae* MAG was conducted based on shared rRNA and ribosomal proteins among 46 and 48 reference genomes, respectively, out of 115 genomes in total, mined from various databases (NCBI, GTDB, and IMG) for uncultivated and cultivated microorganisms identified in the *Verrucomicrobia* phylum (Table S6). The details of these analyses are described in the supplemental material. In addition, we used MiGA (NCBI Prokaryotic taxonomy and the environmental TARA Oceans [Tully] databases; accessed August 2020) and GTDB-Tk (ver. 1.3.0) tools for MAG taxonomic classification.

10.1128/mSphere.00759-21.10 TABLE S6 Conserved markers used in phylogenomic analysis. Integrated microbial genomes (IMG) and/or GenBank accession numbers are listed. Shaded markers were those used for phylogenomic analysis (Fig. 4). Download Table S6, PDF file, 0.1 MB. Copyright © 2021 Murray et al. 2021 Murray et al. https://creativecommons.org/licenses/by/4.0/ This content is distributed under the terms of the Creative Commons Attribution 4.0 International license.

Phylogenetic analysis of the MacB CDS sequences were retrieved from MetatERG annotated *Ca*. Synoicihabitans palmerolidicus contigs, and homologs were retrieved from the NCBI based on BLAST results. Maximum likelihood analysis was conducted on 994 aligned (MUSCLE) positions using RAxML v.8.2.12 using the PROTGAMMALG model and 550 bootstrap replicates. For the phylogenetic analysis of the cellulase CDS, homologs were retrieved from the NCBI based on BLAST results, resulting in 19 sequences. RAxML v.8.2.12 was also used here for maximum likelihood analysis to evaluate the evolutionary relationships based on 496 aligned positions (ClustalOmega) using the PROTGAMMALG model of evolution with 1.000 bootstraps.

### Data availability.

The data (Biosamples and SRA depositions) associated with this study are associated with NCBI BioProject accession no. PRJNA662631. The *Ca*. Synoicihabitans palmerolidicus BioSample identifier (ID) is SAMN18473105, and the genome accession no. is JAGGDC000000000. An annotation data set (.gff) for the *Ca*. Synoicihabitans palmerolidicus MAG resulting from the metaERG pipeline is available (45).
