# Supplementary material for: Discovery of an Antarctic Ascidian-Associated Uncultivated Verrucomicrobia with Antimelanoma Palmerolide Biosynthetic Potential
Source: mSphere. 2021 Dec 1;6(6):e00759-21. doi: 10.1128/mSphere.00759-21 (PMC8636102; doi:10.1128/mSphere.00759-21)
Supplement: TABLE S1 [file msphere.00759-21-st001.pdf]

Metagenome samples	*Synoicum adareanum* lobe ID				Metagenome Assemblies	
	Nor2c-2007	Nor2a-2007	Bon1c-2011	Del2b-2011	Nor2c-2007 and Nor2a2007	Nor2c-2007, Nor2a2007, Bon1c-2011, Del2b-2011
Sequencing Technology	454	Ion Proton	PacBio x 2 lanes	PacBio x 4 lanes	454+Ion Proton	454, Ion Proton, PacBio (Feb 2020)
Raw Reads						
Reads	1,570,137	89,330,870	2,499,689	3,014,737	90,900,996	5,600,821
Total Bases (bp)	904,455,285	17,053,251,055	23,936,900,178	4,127,276,138	17,957,706,340	28,213,247,451
Mean Read Length (bp)	550	190	9575.95	1369.03	197.55	5037.34
Assembly					CoAssembly 1	CoAssembly 2
Number of contigs	-	-	3,485	1,108	86,387	4,215
N50 bp	-	-	42,473	31,254	2,778	40,098
Max contig size bp	-	-	1,040,994	1,419,724	153,680	2,235,039
Min contig size bp	-	-	1,967	426	200	239
total assembly size bp	-	-	88,595,314	20,158,880	144,953,904	97,970,181
