# Supplementary material for: Discovery of an Antarctic Ascidian-Associated Uncultivated Verrucomicrobia with Antimelanoma Palmerolide Biosynthetic Potential
Source: mSphere. 2021 Dec 1;6(6):e00759-21. doi: 10.1128/mSphere.00759-21 (PMC8636102; doi:10.1128/mSphere.00759-21)
Supplement: TABLE S2 [file msphere.00759-21-st002.pdf]

Region	Type	From	To	Most similar cluster	Class	Similarity
12.1	Bacteriocin	11,851	22,753			
16.1	NRPS	1	49,947	nematophin	NRP	25%
92.1	NRPS	1	48,177	Puwainaphycin F / minutissamide B, C, D	NRP	61%
24.1	NRPS, T1PKS	1	41,119	rakicidin A / rakicidin B	NRP:Cyclic depsipeptide+polyketide: modular type 1	22%
27.1	NRPS, T1PKS		41,399			
38.1	NRPS, transAT-PKS-like	1	41,662			
84.1	Terpene	24,732	46,436	hopene	Terpene	23%
87.1	Terpene	20,623	41,480			
72.1	transAT-PKS-like, NRPS	1	42,310	chlorotonil A	Polyketide	15%
78.1	transAT-PKS-like, NRPS	1	68,977	pyxipyrrolone A/ pyxipyrrolone B	NRP + Polyketide	33%
