# Supplementary material for: Discovery of an Antarctic Ascidian-Associated Uncultivated Verrucomicrobia with Antimelanoma Palmerolide Biosynthetic Potential
Source: mSphere. 2021 Dec 1;6(6):e00759-21. doi: 10.1128/mSphere.00759-21 (PMC8636102; doi:10.1128/mSphere.00759-21)
Supplement: TEXT S1 [file msphere.00759-21-t0001.pdf]

Text S1

Materials and Methods

Sample collections. *Synoicum adareanum* lobe samples selected for metagenome sequencing were collected by SCUBA and stored frozen in a buffer (50 mM Tris-HCl at pH 8.0, 50 mM EDTA pH 8.0, 15% sucrose; (1)) at -80 °C until DNA extraction. Samples (Nor-2a-2007 and Nor-2C-2007) were collected from Norsel Point (S 64° 45.638', W 64° 05.874') on 3 May 2007 at a depth of 29 m. Samples Bon1c-2011 and Del2b-2011 were also collected by SCUBA (2) from Bonaparte Point (24 Mar 2011 at 26.2 m) and Delaca Island (28 Mar 2011 at 22 m) and stored frozen (-80 °C) until DNA extraction (see (3) for further sample details).

Sample processing and high molecular weight DNA extraction. For Nor2a-2007 and Nor2C-2007 the outer tissue layer was removed using a scalpel, then ~2.5-gram tissue sections were manually homogenized (1 min.) using a sterile mortar (ice-cold) with a pestle in sterile seawater (12 mL). The cell suspension was sieved through 63 mm sterile Nitex mesh to remove large debris then transferred to a sterile 25 mL Oakridge tube (Nalgene) for centrifugation (300 x g, 15 min at 4 °C) to pellet large cells and debris. The supernatant was then centrifuged at 8000 x g to pellet the bacterial cells at 4 °C. The pellet was resuspended in 200 mL of buffer (50 mM Tris-HCl at pH 8.0, 50 mM EDTA (pH 8.0, 15% sucrose; (1)).

DNA was extracted using the Qiagen Blood and Tissue Kit (Qiagen, Inc.) following manufacturer's instructions. The gDNA was screened by PCR to verify low levels of host (eukaryote) contamination using primers 960F (GGC TTA ATT TGA CTC AAC RCG) and 1200R (5' GGG CAT CAC AGA CCT G 3';(4)). Ketosynthase gene amplification was confirmed in these gDNA extracts following Riesenfeld et al. (5).

Samples Bon-1c-2011 and Del-2b-2011 were collected and homogenized as described by (3), except larger sample sizes were used such that 2.35 and 2.15 grams of tissue (respectively) was homogenized using a MiniLys (Bertin-Instruments,Montigny-le-Bretonneux, France) in a 7 mL tube with 3 mL sterile NaCl (3.5%). High molecular weight gDNA extracted from cell preparations followed Massana et al., (1). Care was taken to preserve the high molecular weight nature of the material e.g., large bore pipet tips, Pasteur pipets used for organic extractions and HMW gDNA pellet was rehydrated at 2 °C overnight gently shaking following ethanol precipitation. These extracts were reprecipitated in 3M NaCl and ethanol (200 proof). Extracts were checked on 0.7 % agarose gels and quantified using a ND-1000 Nanodrop spectrophotometer (Thermo Fisher Scientific, Waltham MA). Subsamples from these same lobes were used for amplicon sequencing (Illumina Inc., San Diego, CA) of the variable 4-5 region of the rRNA gene (3).

454 and Proton metagenome sequencing. 454 pyrosequencing (performed at the Roy J Carver Biotechnology Center, University of Illinois at Urbana-Champaign) was conducted with a bacterial enriched metagenomic DNA preparation from *S. adareanum* lobe (Nor-2c-2007). The MiniLys metagenomic DNA was used to generate single-stranded DNA libraries and emulsion PCR according to established protocols (454 Life Sciences, Roche). Amplified library fragments were sequenced on a Roche Genome Sequencer FLX system initially with a titration sequencing run, followed by a full plate run. Next, an Ion Proton System (Ion Torrent; run at the Nevada

Genomics Center following manufacturer's library preparation and sequencing protocols) was used to sequence a metagenomic DNA sample prepared from *S. adareanum* lobe Nor-2a-2007. See materials and methods in the main manuscript for CoAssembly1 details.

Pacific Biosciences sample preparation and metagenome sequencing details. DNA quality control for samples Bon1c-2011 and Del2b-2011 was confirmed with Qubit Fluorometer (Invitrogen, Inc., Carlsbad CA), NanoDrop 1000 and pulsed field gel electrophoresis (PFGE; BioRad, Hercules, CA). To note, although high molecular weight quality was confirmed on the PFGE, the NanoDrop ratios of 260:230 ratios were 1.2-1.4, much lower than 1.8 which is recommended by Pacific Biosciences (PB; San Diego, CA). The 260/280 ratio improved significantly after two rounds of purification with AMPure® PB beads (initial step in the PB SMRTbell protocol), the 260/230 ratio remained low. gDNA was sheared in G-Tubes (Covaris, Woburn, MA) and purified with 0.45x volume of AMPure PB beads. SMRTbell (PacBio) libraries were prepared according to the PacBio protocol specified in "Procedure and Checklist-Preparing gDNA Libraries Using the SMRTbell Express Template Preparation Kit 2.0". The removal of single-strand overhangs was followed by DNA damage repair reaction, end repair/A-tailing reaction and overhang SMRTbell adapter ligation, with all the steps performed subsequently in one tube. After 0.45x volume AMPure PB purification Bon-1c-2011 and Del-2b-2011 SMRTbell libraries were size selected on Blue Pippin instrument with 6 Kbp and 5 Kbp lower cutoff respectively. Sequencing primer 4 was annealed and DNA polymerase 3.0 was bound to the templates. The libraries were sequenced on a Sequel (PB) with sequencing chemistry 3.0 and 10 or 20hr movies A total of 2 SMRT cells were sequenced for Bon-1c-2011 with a total data output of 23.7 Gb. A total of 4 SMRT cells were sequenced for Del-2b-2011 with a total data output of 4.3 Gb. We obtained 48,298 and 9,576 CCS reads from Bon-1C-2011 and Del-2B-2011, respectively. The average read length was 11,870 bp for Bon-1C-2011 and 10,491 bp for Del-2B-2011 CCS reads.

Binning and bin taxonomic and functional classification. We initially used MaxBin (6) to bin CoAssembly 1 based on the coverage depth, tetranucleotide frequencies and single-copy marker genes, which resulted in 20 "genome"-like bins, containing 63,218 contigs (73.2% of the assembly). CheckM (7) v1.0.11 was used to estimate the genome completeness and potential contamination based on conserved marker genes and perform taxonomic evaluation of the CoAssembly 1 bins using similarity of genomic characteristics, and proximity within a reference genome tree. GTDB-Tk v0.1.3 was also used to evaluate the binned contigs with respect to taxonomic classifications, based on alignment of concatenated marker genes and maximum-likelihood placement within a reference tree, its relative evolutionary divergence, and ANI to reference genomes from GTDB taxonomy database (8).

Initial binning of CoAssembly 1 resulted in 3 bins of interest (Table S3 in which the putative BGC contigs (Fig. 1) that were found in two taxonomically unresolved bins (Bin 1 and Bin 2) dominated by short contigs encoding mostly hypothetical genes with no taxonomic affiliation. Then a third bin was identified (Bin 4, 27 contigs, 143 Kbp) with several contigs attributable to Opitutales, and many additional unclassified contigs. Despite additional re-assemblies, we were not able to link the BGC with these Opitutales scaffolds, thus motivating another round of metagenome sequencing using Pacific Biosciences Sequel Systems technology (PacBio).

CoAssembly 2 contigs were binned using MaxBin2 (9). The bin quality was assessed using CheckM v1.1.2, and GTDB-Tk v1.0.2, and then was used for taxonomic classification of the bins

as above. We implemented a bin-cleaning strategy prior to functional classification of the Opitutaceae bin 8 sequence to reduce errors in classification and assessment of functional properties contributed by contaminating contigs which were evident upon visualization tools provided by MetaERG. This strategy takes a conservative approach (i.e., there is some chance that contigs that were true Verrucomicrobia contigs were discarded), however we felt this was the most robust approach. Examination of those ORFs in contigs discarded suggested 5 out of 24 were classified as having some percentage of ORF assigned to an Opitutaceae genus. First, we used the GTDB-assigned taxonomy as the basis for classification. Next, a custom script was developed to screen contigs in bin 8 with a majority rules algorithm to retain them in the bin if the majority of ORFs on a given contig were assigned to the classified taxonomy. All contigs with the verified identity were placed in the "cleaned" bin (Table S3). The cleaned bin was then run through CheckM v1.1.2 and GTDB-Tk v1.3.0 (10) to evaluate the efficacy of the cleaning algorithm. This effort reduced contamination in the bin, yet retained a similar level of markers identified for Verrucomicrobia. Following manual assembly of the Opitutaceae genome, a re-run with CheckM suggested a number of markers were still absent, these were identified however, through inspection of the MetaERG annotation. Thus, nearly all markers were identified, resulting in a CheckM completeness estimate of 96.04 %. This may have been the result of poor representation of Verrucomicrobia genomes in the CheckM database (12 genomes in the 2015 database that is currently being updated) when doing orthologous searches. When classified using GTDB-Tk the results suggested the closest affiliate was an *Opitutaceae* MAG UBA6669 (the only genome in this un-named genus) – however the result was based on a low average nucleotide identity (75.26) with this medium quality MAG and a low GTDB-Tk alignment fraction (AF) score of 0.02 (calculated as sum of lengths of bidirectional best hits divided by sum of lengths of all genes in each genome separately; AF being most valuable only when circumscribing species).

Real Time PCR. Primer 3 (11), plugin to Geneious (Auckland, NZ) was used to design primers to three coding regions along the candidate palmerolide A BGC (non-ribosomal peptide synthase, acyltransferase, and 3-hydroxymethylglutaryl coenzyme A synthase; amplicon size of 120 bases ea.) for real time PCR following design and optimization criteria recommended by (12). Homology of the primers to sequences other than their targets was evaluated by BLAST to the metagenome assembly. A single GBlocks synthetic positive control was designed with all three gene targets (Integrated DNA Technologies, IDT, Coralville, IA, USA). The GBlocks control also included a 120 base control region matching a putative luciferase CDS (in the putative BGC) that was not used for quantitative assays (Table S5).

As described in the main text, a *S. adareanum* DNA sample set (n=63 *S. adareanum* lobes from 21 colonies) with high levels of palmerolide A were screened with the real time PCR Real time PCR assays on a Quant Studio 3 (Thermo Fisher Scientific, Inc.) at the Nevada Genomics Center. Reactions (15 mL) were run with Power SYBR® Green PCR Master Mix (Applied Biosystems, Thermo Fisher Scientific), following the manufacturer's protocol and thermal cycling conditions (Initial hold at 95 C for 10 minutes followed by 40 cycles of denaturation at 95 °C for 15 sec, and anneal/extension at 60 °C for 1 min. This annealing temperature was confirmed to produce optimal results for the three gene targets at a primer concentration of 0.3 M. Results were analyzed using QuantStudio™ Design and Analysis Software v1.4.3 (Thermo Fisher Scientific) in which gene target copy numbers per ng of DNA template were estimated from standard curves of the synthetic positive control. All reactions had high efficiencies (ave.

99.54, 1.31 s.d., n=13) and r^2 (ave. 0.997, 0.004 s.d., n=13). Pearson correlation coefficients were determined (Microsoft® Excel for Mac v. 16.16.24), then for gene target levels compared to palmerolide A concentrations determined by LC-MS and 16S rRNA gene (variable region 3-4) amplicon sequence variant occurrence levels reported in Murray et al. (3), and the data was plotted using SigmaPlot (v. 14; Systat, San Jose, CA, USA).

Manual MAG assembly and annotation. A manual approach was implemented to arrive at assembly of the *Opitutaceae* MAG of interest. Four bins from different assemblies of the CCS reads, that had 58% GC content (targeted GC percentage of the palmerolide A BGC), were assembled with phrap (overlap based assembler; (13, 14)) and the assembly was visualized with Consed (15). The CCS reads were used to close gaps and verify repetitive elements. A total of ten contigs were resolved, five of which corresponded to sequences that were similar to one another suggesting they were a form of repeated elements within the genome. Rigorous assessment of these repetitive elements, including linking each contig end to other contigs with read-pairing information, assessing estimated gap lengths, and reviewing read coverage along the contigs, strongly suggest that the ten contigs represent the complete genome where each of the five largely unique contig was flanked by contigs corresponding to the repeated elements.

The nature of the five repeated elements that encompass the palmerolide BGC (outlined in Fig. 1) is fully supported by read depth of coverage analysis (e.g., the portions of the palmerolide BGC that are inferred to be present in five copies have 5X the fold coverage of the unique sections of the genome). Due to the very large length of the repeated elements (36.1-73.9 Kbp), no long reads were identified that spanned the entire length of the repeated regions or sufficient amounts that would allow us to specifically order the ten contigs into a single scaffold. A visual representation of the genome in circular format was prepared in GCView (16) in which the five unique contigs, and one possible ordering of the palmerolide BGC repeats is displayed. We used MetaERG (17) and NCBI's PGAP pipeline upon MAG submission (18) as annotation pipelines for analysis of the palmerolide A-containing MAG.

Phylogenomic analyses. We targeted genomes associated with marine and host-associated habitats in the *Opitutaceae* family in addition to including representatives of all *Opitutaceae* genera represented in the GTDB (release 05-RS95). The 115 reference datasets were downloaded from the NCBI and JGI IMG databases. The genome sequences were annotated by Prokka v1.14.5 (19) which performed the open reading frame (ORF) calls and scanned the protein and domain databases in a hierarchical manner from the translated peptide sequences (see Table S6 for list of shared ribosomal proteins and rRNA genes).

Among 115 reference datasets, 62 (many assembled metagenomes and single cell genomes) included 16S rRNA sequences that were identified in the Prokka annotation result. Of these, 47 were unique 16S rRNA sequences without duplication (same genome multiple copy, or identical sequences). The *Opitutaceae* bin 8 (and assembled *Ca*. S. palmerolidicus genome) also included a 16S rRNA identified from the assembled genome. We added the previously sequenced 16S rRNA (FJ169192) and performed the multiple sequence by MUSCLE v3.8.31 (20) and resulting in 49 16S rRNA sequences with 1,636 aligned positions. A maximum likelihood tree was constructed using RAxML v.8.2.12 under the GTRCAT model of evolution and with the number of bootstraps automatically determined (MRE-based bootstopping criterion). A total of 250 bootstrap replicates were conducted under the rapid bootstrapping algorithm, with 100 sampled

to generate proportional support values. The final tree is rooted by *Kiritimatiella* glycovorans L21-Fru-AB and displayed in MegaX (21).

There were 16 ribosomal proteins (*rplB*, *C, D, E, F, N, O, P, and rpsC, H, J, K, L, Q, M, S*) identified shared among 48 reference datasets. Each individual gene set was aligned using MUSCLE. The 16 alignments were concatenated, forming a final alignment comprising 48 genomes and 3,035 amino-acid positions. A maximum likelihood tree was constructed using RAxML v.8.2.12 (22) under the LG plus gamma model of evolution (PROTGAMMALG in the RAxML model section), and with the number of bootstraps automatically determined (MRE-based bootstrapping criterion). A total of 150 bootstrap replicates were conducted under the rapid bootstrapping algorithm, with 100 sampled to generate proportional support values. The final tree is rooted by *Kiritimatiella* glycovorans L21-Fru-AB, a distinct phylum-level lineage originally designated *Verrucomicrobia* (23) and displayed in MegaX.

References

1. Massana R, Taylor LT, Murray AE, Wu KY, Jeffrey WH, DeLong EF. 1998. Vertical distribution and temporal variation of marine planktonic archaea in the Gerlache Strait, Antarctica, during early spring. Limnol Oceanogr 43:607-617.
2. Baker BJ, Dent B. 2020. *Synoicum adareanum* sampling underwater video March 2011 Palmer Station Antarctica, V3. https://doi.org/10.5061/dryad.gxd2547gw doi:https://doi.org/10.5061/dryad.gxd2547gw, Dryad Data.
3. Murray AE, Avalon NE, Bishop L, Davenport KW, Delage E, Dichosa AEK, Eveillard D, Higham ML, Kokkaliari S, Lo C-C, Riesenfeld CS, Young RM, Chain PSG, Baker BJ. 2020. Uncovering the core microbiome and distributions of palmerolide in *Synoicum adareanum* across the Anvers Island archipelago, Antarctica. Mar Drugs 18:298.
4. Gast RJ, Dennett MR, Caron DA. 2004. Characterization of protistan assemblages in the Ross Sea, Antarctica by denaturing gradient gel electrophoresis. Appl Environ Microbiol 70:2028-2037.
5. Riesenfeld CS, Murray AE, Baker BJ. 2008. Characterization of the microbial community and polyketide biosynthetic potential in the palmerolide-producing tunicate, *Synoicum adareanum*. J Nat Prod 71:1812-1818.
6. Wu YW, Tang YH, Tringe SG, Simmons BA, Singer SW. 2014. MaxBin: an automated binning method to recover individual genomes from metagenomes using an expectation-maximization algorithm. Microbiome 2:26.
7. Parks DH, Imelfort M, Skennerton CT, Hugenholtz P, Tyson GW. 2015. CheckM: assessing the quality of microbial genomes recovered from isolates, single cells, and metagenomes. Genome Res 25:1043-1055.
8. Parks D, Chuvochina M, Waite D, Rinke C, Sharshewski A, Chumeil P-A, Hugenholtz P. 2018. A standardized bacterial taxonomy based on genome phylogeny substantially revises the tree of life. Nat Biotechnol 36:996-1004.
9. Wu YW, Simmons BA, Singer SW. 2016. MaxBin 2.0: an automated binning algorithm to recover genomes from multiple metagenomic datasets. Bioinformatics 32:605-607.
10. Chaumeil P-A, Mussig A, Hugenholtz P, Parks D. 2020. GTDB-Tk: a toolkit to classify genomes with the Genome Taxonomy Database. Bioinformatics 36:1925–1927.
11. Untergasser A, Cutcutache I, Koressaar T, Ye J, Faircloth BC, Remm M, Rozen SG. 2012. Primer3-new capabilities and interfaces. Nuc Acids Res 40:e115.

12. Bustin S, Huggett J. 2017. qPCR primer design revisited. Biomolecul Detect Quant 14:19-28.
13. Ewing B, Green P. 1998. Base-calling of automated sequencer traces using phred. II. Error probabilities. Genome Res 8:186-194.
14. Ewing B, Hillier L, Wendl MC, Green P. 1998. Base-calling of automated sequencer traces using phred. I. Accuracy assessment. Genome Res 8:175-185.
15. Gordon D, Abajian C, Green P. 1998. Consed: A graphical tool for sequence finishing. Genome Res 8:195-202.
16. Stothard P, Wishart D. 2005. Circular genome visualization and exploration using GCView. Bioinformatics 21:537-539.
17. Dong XL, Strous M. 2019. An integrated pipeline for annotation and visualization of metagenomic contigs. Front Genetics 10:999.
18. Tatusova T, DiCuccio M, Badretdin A, Chetvernin V, Nawrocki EP, Zaslavsky L, Lomsadze A, Pruitt K, Borodovsky M, Ostell J. 2016. NCBI prokaryotic genome annotation pipeline. Nuc Acids Res 44:6614-6624.
19. Seemann T. 2014. Prokka: rapid prokaryotic genome annotation. Bioinformatics 30:2068-2069.
20. Edgar RC. 2004. MUSCLE: multiple sequence alignment with high accuracy and high throughput. Nuc Acids Res 52:1792-1797.
21. Stecher G, Tamura K, Kumar S. 2020. Molecular Evolutionary Genetics Analysis (MEGA) for macOS. Mol Biol Evol 37:1237-1239.
22. Stamatakis A. 2006. RAxML-VI-HPC: maximum likelihood-based phylogenetic analyses with thousands of taxa and mixed models. Bioinformatics 22:2688-2690.
23. Spring S, Bunk B, Sproer C, Schumann P, Rhode M, Tindall BJ, Klenk H-P. 2016. Characterization of the first cultured representative of Verrucomicrobia subdivision 5 indicates the proposal of a novel phylum. ISME J 10:2801-2816.
