# Supplementary material for: Discovery of an Antarctic Ascidian-Associated Uncultivated Verrucomicrobia with Antimelanoma Palmerolide Biosynthetic Potential
Source: mSphere. 2021 Dec 1;6(6):e00759-21. doi: 10.1128/mSphere.00759-21 (PMC8636102; doi:10.1128/mSphere.00759-21)
Supplement: TABLE S3 [file msphere.00759-21-st003.pdf]

Source	Bin Id	*Ca.* S. palmerolidicus-2	*Ca.* S. palmerolidicus-1	Bin8 - clean CoAssmembly 2	Bin 8 CoAssmembly 2	Bin 4 CoAssembly 1	Bin 1 Coassembly 1	Bin 2 Coassembly 1
GTDB-Tk	Domain	Bacteria	Bacteria	Bacteria	Bacteria	Bacteria	Bacteria	Root (UID1)
	Phylum	Verrucomicrobiota	Verrucomicrobiota	Verrucomicrobiota	Verrucomicrobiota	Verrucomicrobiota	NA	NA
	Classification	Verrucomicrobiae	Verrucomicrobiae	Verrucomicrobiae	Verrucomicrobiae	Verrucomicrobiae	NA	NA
	Order	Opitutales	Opitutales	Opitutales	Opitutales	Opitutales	NA	NA
	Family	Optitutaceae	Optitutaceae	Optitutaceae	Optitutaceae	Optitutaceae	NA	NA
	Genus	UBA6669	UBA6669	UBA6669	UBA6669	NA	NA	NA
CheckM	# genomes	88	88	88	88	88	5449	5656
	# markers	227	227	230	230	230	104	56
	# marker sets	146	146	148	148	148	58	24
	0	7	51	35	34	197	103	56
	1	218	174	184	169	33	1	0
	2	1	1	10	25	0	0	0
	3	0	0	1	2	0	0	0
	4	0	0	0	0	0	0	0
	5	1	1	0	0	0	0	0
	Completeness	96.04	71.89	80.86	82.04	4.59	1.72	0
	Contamination	3.42	3.42	4.62	8.43	0	0	0
	Strain heterogeneity	100	100	76.92	35.48	0	0	0
GTDB-Tk	closest_placement_reference	NA	GCA_002455175.1	GCA_002321895.1	NA	NA	NA	NA
	closest_placement_radius	NA	95	95	NA	NA	NA	NA
	closest_placement_taxonomy - species	NA	UBA6669 sp002455175	UBA6669 sp002455175	NA	NA	NA	NA
	closest_placement_ani	NA	75.26	75.16	NA	NA	NA	NA
	closest_placement_af	NA	0.02	0.02	NA	NA	NA	NA
	classification_method	NA	taxonomic classification defined by topology and ANI	taxonomic classification defined by topology and ANI	NA	NA	NA	NA
	aa_percent	NA	67.3	56.53	61.11	NA	NA	NA
	red_value	NA	0.920899756	0.920064441	0.910022563	NA	NA	NA
