# Supplementary figures and images for: Discovery of an Antarctic Ascidian-Associated Uncultivated Verrucomicrobia with Antimelanoma Palmerolide Biosynthetic Potential
Source: mSphere. 2021 Dec 1;6(6):e00759-21. doi: 10.1128/mSphere.00759-21 (PMC8636102; doi:10.1128/mSphere.00759-21)

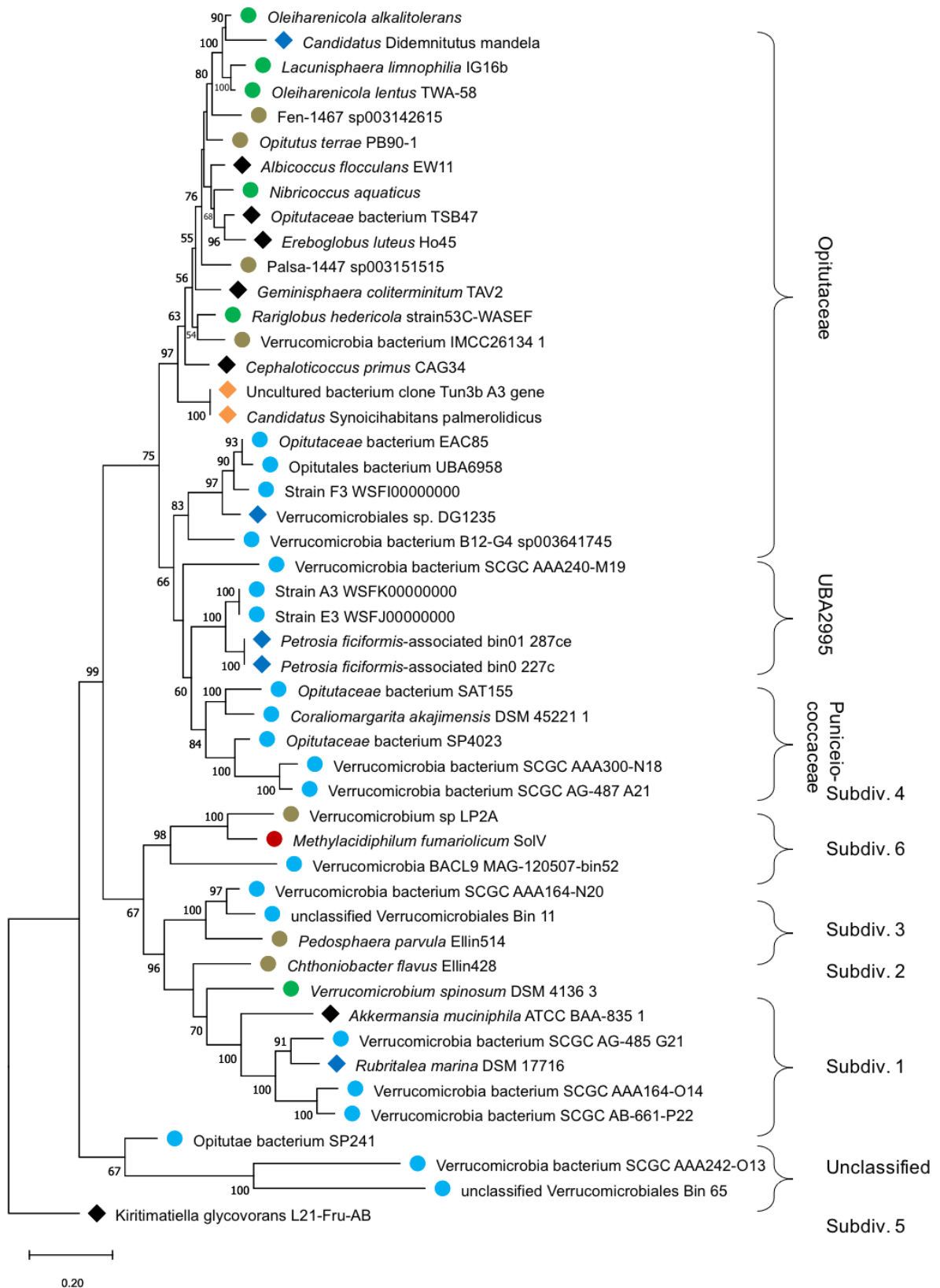

Supplement: FIG S1 [file msphere.00759-21-sf001.pdf]

A

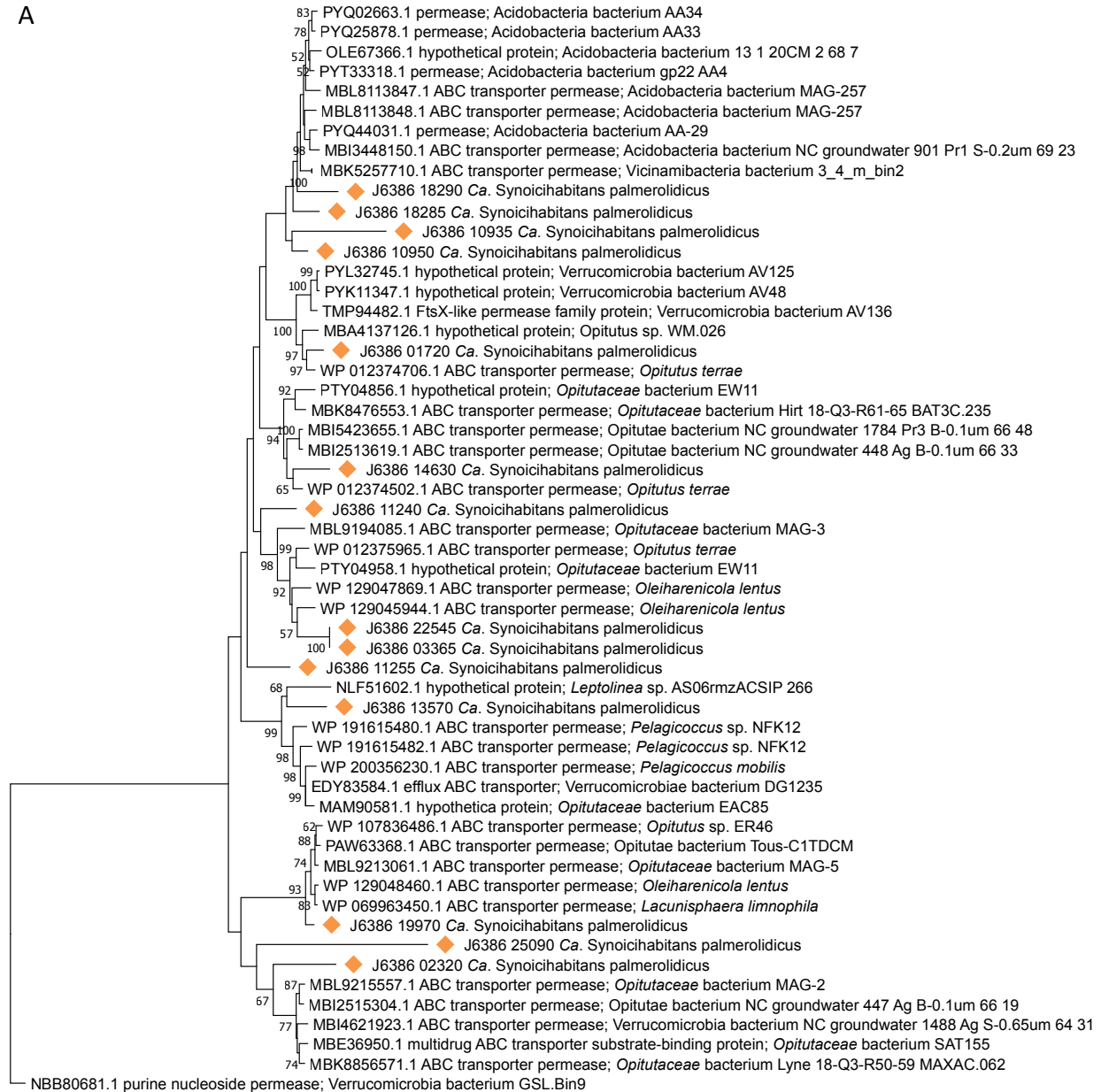

B

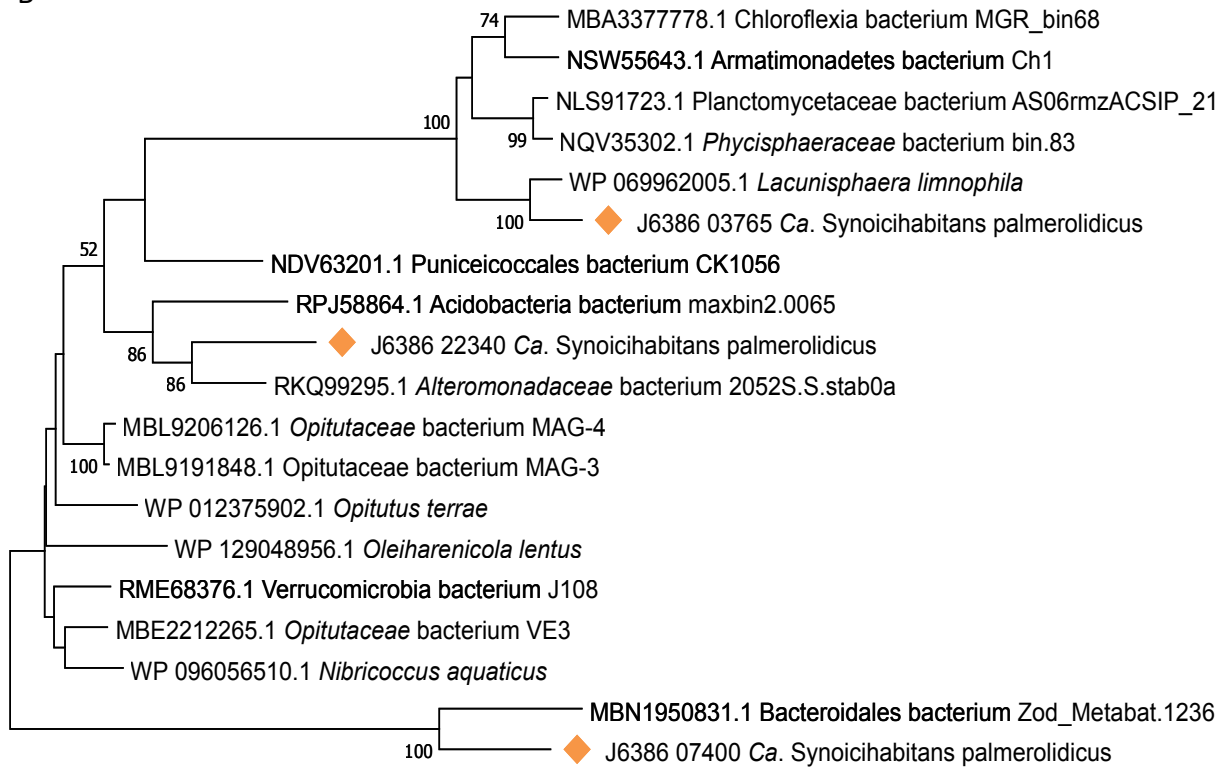

0.50

Supplement: FIG S2 [file msphere.00759-21-sf002.pdf]

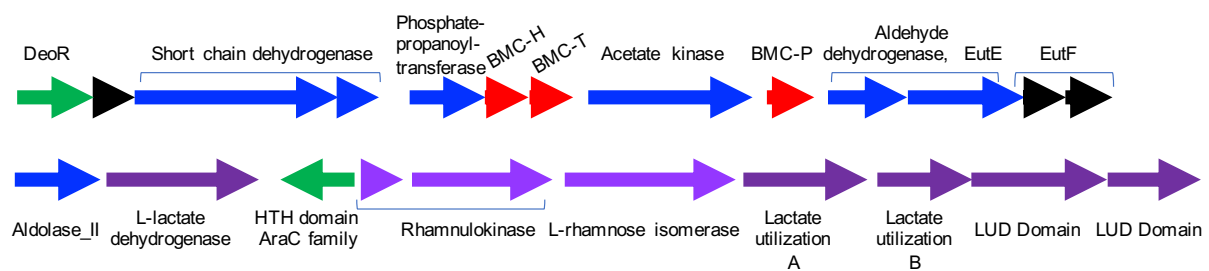

Supplement: FIG S3 [file msphere.00759-21-sf003.pdf]
