# Supplementary material for: Discovery of an Antarctic Ascidian-Associated Uncultivated Verrucomicrobia with Antimelanoma Palmerolide Biosynthetic Potential
Source: mSphere. 2021 Dec 1;6(6):e00759-21. doi: 10.1128/mSphere.00759-21 (PMC8636102; doi:10.1128/mSphere.00759-21)
Supplement: TABLE S4 [file msphere.00759-21-st004.pdf]

Species	Strain	Genome accession	% GC
Cephaloticoccus capnophilus	CV41	RS_GCF_001580045.1	60.54%
Cephaloticoccus primus	CAG34	RS_GCF_001580015.1	63.03%
Cephalitcoccus sp002367815*		GB_GCA_002367815.1	54.25%
Cephalitcoccus sp002713695*		GB_GCA_002713695.1	55.96%
Cephalitcoccus sp002713695*		GB_GCA_002471335.1	54.13%
Ca. Didemnitutus mandela*		GB_GCA_002591725.1	51.93%
Ereboglobus luteus	Ho45	RS_GCF_003096195.18	59.67%
Ereboglobus sp001650175	TSB47	RS_GCF_001650175.1	62.88%
Geminisphaera colitermitum	TAV2	RS_GCF_000171235.2	60.93%
Geminisphaera colitermitum	TAV3	RS_GCF_001653815.2	60.89%
Geminisphaera colitermitum	TAV4	RS_GCF_001653825.2	60.85%
Geminisphaera sp000242935	TAV5	RS_GCF_000242935.2	63.45%
Geminisphaera sp000242935	TAV1	RS_GCF_000243495.5	63.25%
Lacunisphaera limnophila	IG16b	RS_GCF_001746835.1	66.48%
Lacunisphaera sp001464505		GB_GCA_001464505.1	64.27%
Lacunisphaera sp002304425		GB_GCA_002304425.1	56.80%
Lacunisphaera sp004118375	TWA-58	GB_GCA_004118375.1	65.30%
Lacunisphaera sp005787865		GB_GCA_005787865.1	56.64%
Lacunisphaera sp900104025	GAS368	GB_GCA_900104025.1	65.67%
Nibricoccus aquaticus	HZ-65	RS_GCF_002310495.1	65.25%
Opitutus terrae	PB90-1	RS_GCF_000019965.1	65.34%
Opitutus sp002288455		GB_GCA_002288455.1	67.69%
Opitutus sp002304435		GB_GCA_002304435.1	69.98%
Opitutus sp003054705	ER46	GB_GCA_003054705.1	67.07%
Opitutus sp005786775		GB_GCA_005786775.1	70.04%
Opitutus sp005792355		GB_GCA_005792355.1	64.74%
Opitutus sp005792355		GB_GCA_005779485.1	64.83%
B12-G4 sp003641745		GB_GCA_003641745.1	58.28%
DG1235 sp000155695	DG1235	RS_GCF_000155695.1	54.26%
EW11 sp003054665	EW11	RS_GCF_003054665.1	63.63%
Fen-1467 sp003142615		GB_GCA_003142615.1	64.26%
Fen-1467 sp003142615		GB_GCA_003141355.1	63.94%
Fen-1467 sp003142615		GB_GCA_003142335.1	64.50%
Fen-1467 sp003142955		GB_GCA_003142955.1	62.41%
Fen-1467 sp003142955		GB_GCA_003157795.1	62.27%
Fen-1467 sp003142955		GB_GCA_003154875.1	62.37%
Fen-1467 sp003153035		GB_GCA_003153035.1	64.04%
Fen-1467 sp003153035		GB_GCA_003160855.1	64.04%
Fen-1467 sp003153035		GB_GCA_003157495.1	64.27%
Fen-1467 sp003157575		GB_GCA_003157575.1	63.34%
IMCC26134 sp002382525		GB_GCA_002382525.1	63.52%
IMCC26134 sp002382525		GB_GCA_002383805.1	63.56%
IMCC26134_A sp000972765	IMCC26134	RS_GCF_000972765.1	61.28%
J108 sp003694825		GB_GCA_003694825.1	65.52%
Opi-474 sp003402695		GB_GCA_003402695.1	65.60%
Palsa-1447 sp003140665		GB_GCA_003140665.1	66.23%

Species	Strain	Genome accession	% GC
Palsa-1447 sp003140665		GB_GCA_003133585.1	66.47%
Palsa-1447 sp003140665		GB_GCA_003167995.1	66.10%
Palsa-1447 sp003140665		GB_GCA_003167115.1	66.40%
Palsa-1447 sp003151495		GB_GCA_003151495.1	66.51%
Palsa-1447 sp003151515		GB_GCA_003151515.1	67.03%
Palsa-1447 sp003164715		GB_GCA_003164715.1	66.99%
Palsa-1447 sp003168315		GB_GCA_003168315.1	66.69%
Tous-C4FEB sp002304445		GB_GCA_002304445.1	62.76%
Tous-C4FEB sp002304445		GB_GCA_002288405.1	62.94%
Tous-C4FEB sp002304445		GB_GCA_005789435	62.94%
Tous-C4FEB sp002737275		GB_GCA_002737275.1	62.84%
Tous-C4FEB sp005799525		GB_GCA_005799525.1	63.21%
UBA2377 sp002344205		GB_GCA_002344205.1	65.29%
UBA2377 sp002344205		GB-GCA_002343065.1	65.24%
UBA5691 sp002420185*		GB_GCA_002320185.1	48.68%
UBA5691 sp002420265*		GB_GCA_002420265.1	49.76%
UBA5691 sp002420265*		GB_GCA_002722675.1	49.75%
UBA5691 sp002424265*		GB_GCA_002170515.2	49.90%
UBA5691 sp002474325*		GB_GCA_002474325.1	50.38%
UBA5691 sp002474325*		GB_GCA_002450395.1	50.42%
UBA4691 sp002694885*		GB_GCA_002694885.1	51.27%
UBA4691 sp002694885*		GB_GCA_003525105.1	51.45%
UBA6669 sp002455175		GB_GCA_002455175.1	57.04%
		average	61.58%
		stdev	0.06
