# Supplementary material for: Discovery of an Antarctic Ascidian-Associated Uncultivated Verrucomicrobia with Antimelanoma Palmerolide Biosynthetic Potential
Source: mSphere. 2021 Dec 1;6(6):e00759-21. doi: 10.1128/mSphere.00759-21 (PMC8636102; doi:10.1128/mSphere.00759-21)
Supplement: TABLE S5 [file msphere.00759-21-st005.pdf]

Gene Target	Primer ID	Sequence
NRPS Condensation domain (NRPS)	PalA-NRPS _ 531F	5'-CGCCAAGTTGGATCGAGACT-3'
	PalA-NRPS _ 650R*	5'-GAGCGATTGGTGATTCCGGA-3'
Acyl transferase (AT) #	PalA-AT-1_624F	5'-CACCGCACTACCCCATGAAT-3'
	PalA-AT-1_743R*	5'-CAGACGGAACCTGAGTTCGT-3'
3'hydroxymethylglutaryl synthase (HCS)	PalA-HCS_989F	5'-ACTGGGTATTCGGCGTGAAG-3'
	PalA-HCS_1108R*	5'-GGTGCGTAACTACCATCGGG-3'

† GBlocks synthetic positive qPCR control sequence in which the NRPS positions 531-650 in the CDS are highlighted in yellow, luciferase positions 589-708 are highlighted in gray (not used in this study), AT1 positions 624-743 are highlighted in light blue and HCS are highlighted in green.

5’ATGTACTTGGAATCCGACACTTTTTTCGCCAAGTTGGATCGAGACTACTGGTTGCAGCGTTTCCCCAAGGGGTTCCAGCCCGTGTTTCCCGCGAATGGCGATATTAAGGACTCCGGCGGGGAGATGGAGCGATTGGTGATTCCGGATTTTTTTCGACCTTGGAAGAACTCGCTAAAAAAATTGCGCGTTACCGTGCTGCACGGGCTGAGGCGGGCCTGGATCCGGTGGGAGGGTGTGTGACTCTCATGCTGCACACGTTTGTGGATCCCGATTTTTTTCACCGCACTACCCCATGAATCGACGACGCTAAAACAGATGTTGGCGCGGCAAATTACGAGCCCGGTGCGATGGACAGAGACCATGCGCTGGCTGTTTCGGCAGACGGAACCTGAGTTCGTTTTTTTTACTGGGTATTCGGCGTGAAGGATCAGGAAGTGGACTTAGCTCCTTACCGAAACCTCTACGACCAAACCTTGGCGGGCCGTGGTTTGTTGGTGTTGAAAGGGGTGCGTAACTACCATCGGGAATATGACTGGAGTTGATAA 3’

* Reverse sequences are not reverse and complimented.

# The acyltransferase domain targeted was the first of two domains in the putative *pal* BGC as oriented with 5' being the NRP at the beginning of the cluster.
