# Supplementary material for: Discovery of an Antarctic Ascidian-Associated Uncultivated Verrucomicrobia with Antimelanoma Palmerolide Biosynthetic Potential
Source: mSphere. 2021 Dec 1;6(6):e00759-21. doi: 10.1128/mSphere.00759-21 (PMC8636102; doi:10.1128/mSphere.00759-21)
Supplement: TABLE S6 [file msphere.00759-21-st006.pdf]

IMG ID	GenBank Accession	Taxon ID	infB	lepA	pheS	rplB	rplC	rplD	rplE	rplF	rplK	rplN	rplO	rplP	rpsB	rpsC	rpsE	rpsG	rpsH	rpsI	rpsJ	rpsK	rpsL	rpsM	rpsQ	rpsS	16S rRNA
		Candidatus Synoicohabitans palmerolidicus	+	+	+	+	+	+	+	+	+	+	+	+	+	+	+	+	+	+	+	+	+	+	+	+	+
647533243	GCA_000155695	*Verrucomicrobiales* sp. DG1235	+	+	+	+	+	+	+	+	+	+	+	+	+	+	+	+	+	+	+	+	+	+	+	+	+
2228664034	GCA_000383755	Verrucomicrobia bacterium SCGC AAA300-N18 (unscreened)	+	+	-	+	+	+	+	-	-	+	-	+	-	+	-	+	+	-	+	-	+	-	+	+	+
2228664038	GCA_000385315	Verrucomicrobia bacterium SCGC AAA164-P11 (unscreened)	-	-	-	-	-	-	-	-	-	-	-	-	-	-	-	-	-	-	-	-	-	-	-	-	+
2228664042	GCA_000385235	Verrucomicrobia bacterium SCGC AAA164-A08 (unscreened)	-	-	-	-	-	-	-	-	-	-	-	-	-	-	-	-	-	-	-	-	-	-	-	-	-
2228664043	GCA_000385255	Verrucomicrobia bacterium SCGC AAA164-B23 (unscreened)	-	-	-	-	-	-	-	-	-	-	-	-	-	-	-	-	-	-	-	-	-	-	-	-	-
2228664044	GCA_000385275	Verrucomicrobia bacterium SCGC AAA164-I21 (unscreened)	-	-	+	+	+	+	+	+	-	+	+	+	-	+	+	+	+	-	+	+	+	+	+	+	+
2228664050	GCA_000383735	Verrucomicrobia bacterium SCGC AAA164-M04 (unscreened)	-	-	-	-	-	-	-	-	+	-	-	-	+	-	-	-	-	+	-	-	-	-	-	-	-
2228664051	GCA_000385295	Verrucomicrobia bacterium SCGC AAA164-N20 (unscreened)	-	-	-	+	+	+	+	+	-	+	+	+	-	+	+	+	+	-	+	+	+	+	+	+	+
2236347002	GCA_000383715	Verrucomicrobia bacterium SCGC AAA164-E04 (unscreened)	+	-	+	+	+	+	+	+	+	+	+	+	-	+	+	+	+	+	+	+	+	+	+	+	+
2236347003	GCA_000264625	Verrucomicrobia bacterium SCGC AAA168-E21	+	-	+	-	-	-	-	-	+	-	-	-	-	-	-	-	-	-	-	-	-	-	-	-	+
2236347021	GCA_000264645	Verrucomicrobia bacterium SCGC AAA168-F10	+	-	+	+	+	+	+	+	+	-	+	+	-	+	-	+	-	-	+	+	+	+	+	+	+
2236661017	GCA_000382665	Verrucomicrobia bacterium SCGC AAA300-K03 (unscreened)	-	-	-	+	+	+	+	+	+	+	+	+	+	+	+	+	+	-	+	+	+	+	+	+	+
2236661018	GCA_000382685	Verrucomicrobia bacterium SCGC AAA300-O17 (unscreened)	-	-	-	+	+	+	+	+	+	+	+	+	+	+	+	+	+	-	+	+	+	+	+	+	-
2517572138	GCA_000264585	Verrucomicrobia bacterium SCGC AAA164-A21 (unscreened)	-	-	-	-	-	-	-	-	-	-	-	-	-	-	-	-	-	-	-	-	-	-	-	-	-
2517572141	GCA_000264625	Verrucomicrobia bacterium SCGC AAA168-E21 (unscreened)	+	-	+	-	-	-	-	-	+	-	-	-	-	-	-	-	-	-	-	-	-	-	-	-	+
2517572140	GCA_000264605	Verrucomicrobia bacterium SCGC AAA164-O14 (unscreened)	-	+	+	+	+	+	+	+	-	+	-	+	+	+	+	+	+	-	+	-	+	-	+	+	+
2517572142		Verrucomicrobia bacterium SCGC AAA168-F10 (unscreened)	+	-	+	-	-	-	-	-	+	-	-	-	-	-	-	-	-	-	-	-	-	-	-	-	+
2616645036		*D. pulchra* _bleached_metagenome_bin377	+	+	+	+	+	+	+	+	+	+	+	+	+	+	+	+	+	+	+	+	+	+	+	+	-
2634166657		Verrucomicrobia bacterium SCGC AC-661-N10 (unscreened)	-	+	-	-	-	-	-	-	-	-	-	-	-	-	-	-	-	-	-	-	-	-	-	-	+
2634166686		Verrucomicrobia bacterium SCGC AD-105-D22 (unscreened)	-	-	+	+	-	-	+	+	-	+	+	+	-	+	+	-	+	-	-	+	-	+	+	+	+
2634166709		Verrucomicrobia bacterium SCGC AD-265-E10 (unscreened)	-	-	-	-	-	-	-	-	-	-	-	-	-	-	-	-	-	-	-	-	-	-	-	-	-
2634166715		Verrucomicrobia bacterium SCGC AAA240-M19 (unscreened)	+	+	+	+	-	+	+	+	-	+	+	+	+	+	+	-	+	+	-	+	-	+	+	+	+
2634166718		Verrucomicrobia bacterium SCGC AAA242-O13 (unscreened)	-	-	-	-	-	-	-	-	-	-	-	-	-	-	-	-	-	-	-	-	-	-	-	-	+
2634166725		Verrucomicrobia bacterium SCGC AB-606-A23 (unscreened)	-	-	-	+	+	+	+	+	-	+	+	+	-	+	-	+	+	-	+	+	+	+	+	+	-
2634166742		Verrucomicrobia bacterium SCGC AB-661-L11 (unscreened)	-	-	-	-	-	-	-	-	-	-	-	-	-	-	-	-	-	-	-	-	-	-	-	-	-
2634166743		Verrucomicrobia bacterium SCGC AB-661-P22 (unscreened)	-	-	-	-	-	-	-	-	+	-	-	-	+	-	-	-	-	-	-	-	-	-	-	-	+
2634166791		Verrucomicrobia bacterium SCGC AC-312-P03 (unscreened)	-	-	-	-	-	-	-	-	-	-	-	-	-	-	-	-	-	-	-	-	-	-	-	-	-
2651870083		unclassified Verrucomicrobiales Bin 11	+	+	-	-	-	-	-	-	-	-	-	-	+	-	-	-	-	+	-	+	-	+	-	-	+
2651870084		unclassified Verrucomicrobiales Bin 46	-	-	-	-	-	-	-	-	+	-	-	-	+	-	-	-	-	+	-	+	-	+	-	-	-
2651870085		unclassified Verrucomicrobiales Bin 30	-	-	-	-	-	-	+	+	-	-	+	-	-	-	+	+	+	-	-	+	+	+	-	-	-
2651870086		unclassified Verrucomicrobiales Bin 34	+	-	-	-	-	-	+	+	-	+	+	+	+	+	+	+	+	-	-	+	+	+	+	-	-
2651870087		unclassified Verrucomicrobiales Bin 65	-	-	-	+	-	-	-	-	-	+	-	+	-	+	-	-	-	-	-	-	-	-	+	+	+
2657245246		Verrucomicrobia bacterium SCGC AC-312_D05v2 (unscreened)	-	-	-	-	-	-	-	-	-	-	-	-	-	-	-	-	-	-	-	-	-	-	-	-	+
2657245247		Verrucomicrobia bacterium SCGC AC-312_D05v2 (contamination screened)	-	-	-	-	-	-	-	-	-	-	-	-	-	-	-	-	-	-	-	-	-	-	-	-	+
2657245291		Verrucomicrobia bacterium SCGC AC-337_K21 - v2 (unscreened)	-	-	-	-	-	-	-	-	-	-	-	-	-	-	-	-	-	-	-	-	-	-	-	-	+
2657245292		Verrucomicrobia bacterium SCGC AC-337_K21 - v2 (contamination screened)	-	-	-	-	-	-	-	-	-	-	-	-	-	-	-	-	-	-	-	-	-	-	-	-	+
2657245323		Verrucomicrobia bacterium SCGC AC-312_O22 - v2 (unscreened)	-	+	-	-	-	-	-	-	-	-	-	-	-	-	-	-	-	-	-	-	-	-	-	-	+
2657245324		Verrucomicrobia bacterium SCGC AC-312_O22 - v2 (contamination screened)	-	-	-	-	-	-	-	-	-	-	-	-	-	-	-	-	-	-	-	-	-	-	-	-	-
2663762693		Verrucomicrobia bacterium SCGC AC-312_N19v2 (unscreened)	-	+	-	-	-	-	-	-	-	-	-	-	-	-	-	-	-	-	-	-	-	-	-	-	+
2684622978		Verrucomicrobia bacterium SCGC AG-542-N08	-	-	+	-	-	-	-	-	-	-	-	-	+	-	-	-	-	-	-	-	-	-	-	-	+
2684622991		Verrucomicrobia bacterium SCGC AG-542-I16	+	-	-	-	-	-	-	-	-	-	-	-	-	-	-	-	-	-	-	-	-	-	-	-	-
2693429924		Verrucomicrobia bacterium SCGC AC-337_A14 - v2 (unscreened)	-	-	-	-	-	-	-	-	-	-	-	-	-	-	-	-	-	-	-	-	-	-	-	-	+
2693429925		Verrucomicrobia bacterium SCGC AC-337_A14 - v2 (contamination screened)	-	-	-	-	-	-	-	-	-	-	-	-	-	-	-	-	-	-	-	-	-	-	-	-	+
2693429980		Verrucomicrobia bacterium SCGC AC-337_J20 - v2 (unscreened)	-	-	-	-	-	-	-	+	-	-	+	-	-	-	+	-	+	-	-	+	-	+	-	-	-
2693429981		Verrucomicrobia bacterium SCGC AC-337_J20 - v2 (contamination screened)	-	-	-	-	-	-	-	-	-	-	-	-	-	-	-	-	-	-	-	+	-	-	-	-	-
2695420979		unclassified Verrucomicrobiales bin 1	-	-	+	+	+	+	+	+	-	-	+	-	+	-	+	-	+	+	+	+	-	-	-	+	-
2695420980		unclassified Verrucomicrobiales bin 2	-	-	-	+	-	-	-	-	-	-	-	-	+	-	-	-	-	-	-	-	-	-	-	+	-

IMG ID	GenBank Accession	Taxon ID	infB	lepA	pheS	rplB	rplC	rplD	rplE	rplF	rplK	rplN	rplO	rplP	rpsB	rpsC	rpsE	rpsG	rpsH	rpsI	rpsJ	rpsK	rpsL	rpsM	rpsQ	rpsS	16S rRNA
2700988675		Verrucomicrobia bacterium SCGC AC-337_A09v3 (contamination screened)	-	-	-	-	-	-	-	-	-	-	-	-	-	-	-	-	-	-	-	-	-	-	-	-	+
2731639125		Verrucomicrobia bacterium SCGC AG-485_L14 (contamination screened)	-	-	+	-	-	-	-	-	-	-	-	-	-	-	-	-	-	-	-	-	-	-	-	-	-
2731639127		Verrucomicrobia bacterium SCGC AG-485_G21 (contamination screened)	-	-	-	+	+	+	+	-	+	+	+	+	-	+	+	+	+	-	+	+	+	+	+	+	+
2731639129		Verrucomicrobia bacterium SCGC AG-487_A21 (contamination screened)	-	-	+	-	-	-	-	-	-	-	-	-	+	-	-	-	-	-	-	-	-	-	-	-	+
2731639131		Verrucomicrobia bacterium SCGC AG-487_G17 (contamination screened)	+	-	-	-	-	-	-	-	-	-	-	-	-	-	-	-	-	-	-	-	-	-	-	-	+
2739367775		Verrucomicrobiales bacterium JGI 01_E13 (contamination screened)	-	+	-	+	+	+	-	-	+	-	-	-	-	-	-	+	-	-	+	-	+	-	-	+	-
2786546549	GCA_002726435	Opitutae bacterium NP78	-	-	+	+	+	+	+	+	-	+	+	+	-	+	+	+	+	+	+	+	+	+	+	+	-
2786546550	GCA_002716045	Opitutae bacterium SP85	+	+	+	+	+	+	+	+	+	+	+	+	-	+	+	+	+	+	+	+	+	+	+	+	-
2786546551	GCA_002722545	Opitutae bacterium SP216	+	+	+	+	+	+	+	+	+	+	+	+	+	+	+	+	+	+	+	+	+	+	+	+	-
2786546552	GCA_002721705	Opitutae bacterium SP241	+	+	+	+	+	+	+	+	+	+	+	+	+	+	+	+	+	+	+	+	+	+	+	+	+
2786546553	GCA_002713685	Opitutae bacterium SAT156	+	+	-	+	+	+	+	+	-	+	+	+	+	+	+	+	+	-	+	+	+	+	+	+	-
2786546554	GCA_002730975	Opitutae bacterium NP117	+	+	+	+	+	+	+	+	+	+	+	+	-	+	+	+	+	+	+	+	+	+	+	+	-
2786546555	GCA_002695645	Opitutae bacterium EAC647	+	+	+	+	+	+	+	+	+	+	+	+	-	+	+	+	+	-	+	+	+	+	+	+	-
2786546556	GCA_002686835	Opitutae bacterium ARS76	-	+	+	+	+	+	+	+	+	+	+	+	+	+	+	+	+	+	+	+	+	+	+	+	-
2786546557	GCA_002689665	Opitutae bacterium ARS1007	-	+	-	+	+	+	+	+	+	+	+	+	+	+	+	+	+	+	+	+	+	+	+	+	-
2786546558	GCA_002692605	Opitutae bacterium IN42	+	+	-	+	+	+	+	+	+	+	+	+	+	+	+	+	+	+	+	+	+	+	+	+	-
2786546559	GCA_002702485	Opitutae bacterium NAT243	-	-	-	+	+	+	+	+	+	+	+	-	-	+	+	+	+	+	+	+	+	+	+	+	-
2786546560	GCA_002694035	Opitutae bacterium IN48	+	+	+	+	+	+	+	+	+	+	+	+	+	+	+	+	+	+	+	+	+	+	+	+	-
2786546561	GCA_002692535	Opitutae bacterium IN921	+	+	-	-	-	-	+	+	+	+	+	+	+	+	+	-	+	+	-	+	-	+	+	+	-
2786546708	GCA_002725655	Opitutales bacterium NP990	-	+	+	+	+	+	+	+	+	+	+	+	-	+	+	+	+	-	+	+	+	+	+	+	-
2786546709	GCA_002724235	Opitutales bacterium RS400	+	+	+	-	-	-	-	-	-	-	-	-	-	-	-	-	-	+	-	-	-	-	-	-	-
2786546710	GCA_002720915	Opitutales bacterium SP2995	+	+	+	+	+	+	-	-	-	-	-	+	+	+	-	-	-	+	-	-	-	-	-	+	-
2786546935	GCA_002713695	*Opitutaceae* bacterium SAT155	+	+	+	+	+	+	+	+	+	+	+	+	+	+	+	+	+	+	+	+	+	+	+	+	+
2786546936	GCA_002694885	*Opitutaceae* bacterium EAC85	+	+	-	+	+	+	+	+	+	+	+	+	+	+	+	+	+	+	+	+	+	+	+	+	+
2786546938	GCA_002722675	*Opitutaceae* bacterium SP211	+	+	-	+	+	+	+	+	-	+	+	+	-	+	+	+	+	+	+	-	+	+	-	+	-
2786546939	GCA_002717045	*Opitutaceae* bacterium SP4023	+	+	+	+	+	+	+	+	+	+	+	+	+	+	+	+	+	+	+	-	+	+	+	+	+
	GCA_002591725	*Candidatus* Didemnitutus mandela	+	+	+	+	+	+	+	+	+	+	+	+	+	+	+	+	+	-	+	+	+	+	+	+	+
	GCA_003525105	Opituae bacterium UBA10075	+	-	+	+	+	+	-	-	-	-	-	+	+	+	-	+	-	-	+	+	+	+	+	+	-
	GCA_003482665	*Opitutaceae* bacterium UBA8745	+	-	-	+	+	+	+	+	-	+	-	+	-	+	-	+	+	-	+	+	+	+	+	+	-
	GCA_002420265	Opitutales bacterium UBA5691	+	+	+	+	+	+	+	+	+	+	+	+	-	+	+	-	+	+	+	+	-	+	+	+	-
	GCA_002420185	Opitutales bacterium UBA5694	+	+	+	+	+	+	+	+	+	+	+	+	+	+	+	+	+	+	+	+	+	+	+	+	-
	GCA_002450395	Opitutales bacterium UBA6958	+	+	+	+	+	+	+	+	+	+	+	+	-	+	+	+	+	+	+	+	+	+	+	+	+
	GCA_002474325	Opitutales bacterium UBA7389	+	+	+	+	+	+	+	+	+	+	+	+	+	+	+	+	+	+	+	+	+	+	+	+	-
	GCA_002367815	*Opitutaceae* bacterium UBA3033	+	-	+	+	+	+	+	+	-	+	+	+	+	+	+	+	+	+	+	+	+	+	+	+	-
	GCA_002471335	*Opitutaceae* bacterium UBA7327	+	+	+	+	+	+	+	+	+	+	+	+	+	+	+	+	+	+	+	+	+	+	+	+	-
	GCA_000019965	*Opitutus terrae* PB90-1	+	+	+	+	+	+	+	+	+	+	+	+	+	+	+	+	+	+	+	+	+	+	+	+	+
	GCA_000025905	*Coraliomargarita akajimensis* DSM 45221	+	+	+	+	+	+	+	+	+	+	+	+	+	+	+	+	+	+	+	+	+	+	+	+	+
	GCA_000171235	*Geminisphaera coliterminitum* TAV2	+	+	+	+	+	+	+	+	+	+	+	+	+	+	+	+	+	+	+	+	+	+	+	+	+
	GCA_000172555	*Pedosphaera parvula* Ellin514	+	+	+	+	+	+	+	+	+	+	+	+	+	+	+	+	+	+	+	+	+	+	+	+	+
	GCA_000173075	*Chthoniobacter flavus* Ellin428	+	+	+	+	+	+	+	+	+	+	+	+	+	+	+	+	+	+	+	+	+	+	+	+	+
	GCA_000378105	*Rubritalea marina* DSM 17716	+	+	+	+	+	+	+	+	+	+	+	+	+	+	+	+	+	+	+	+	+	+	+	+	+
	GCA_000972765	Verrucomicrobia bacterium IMCC26134	+	+	+	+	+	+	+	+	+	+	+	+	+	+	+	+	+	+	+	+	+	+	+	+	+
	GCA_001017655	*Kiritimatiella glycovorans* L21-Fru-AB	+	+	+	+	+	+	+	+	+	+	+	+	+	+	+	+	+	+	+	+	+	+	+	+	+
	GCA_001438005	Verrucomicrobia BACL9 MAG-120507-bin52	+	+	-	+	+	+	+	+	+	+	+	+	+	+	+	+	+	+	+	+	+	+	+	+	+
	GCA_001580015	*Cephaloticoccus primus* CAG34	+	+	+	+	+	+	+	+	+	+	+	+	+	+	+	+	+	+	+	+	+	+	+	+	+
	GCA_001650175	*Opitutaceae* bacterium TSB47	+	+	+	+	+	+	+	+	+	+	+	+	+	+	+	+	+	+	+	+	+	+	+	+	+
	GCA_001746835	*Lacunisphaera limnophilia* IG16b	+	+	+	+	+	+	+	+	+	+	+	+	+	+	+	+	+	+	+	+	+	+	+	+	+
	GCA_000020225	*Akkermansia muciniphila* ATCC BAA-835	+	+	+	+	+	+	+	+	+	+	+	+	+	+	+	+	+	+	+	+	+	+	+	+	+
	GCA_000172155	*Verrucomicrobium spinosum* DSM 4136	+	+	+	+	+	+	+	+	+	+	+	+	+	+	+	+	+	+	+	+	+	+	+	+	+
	GCA_000526255	*Verrucomicrobium* sp. LP2A	+	+	+	+	+	+	+	+	+	+	+	+	+	+	+	+	+	+	+	+	+	+	+	+	+
	GCA_000953475	*Methylacidiphilum fumariolicum* SolV	+	+	+	+	+	+	+	+	+	+	+	+	+	+	+	+	+	+	+	+	+	+	+	+	+
2786546940	GCA_003054665	*Albicoccus flocculans* EW11	-	+	+	+	+	+	+	+	+	+	+	+	-	+	+	+	+	+	+	+	+	+	-	+	+
	GCA_002455175	*Opitutaceae* bacterium UBA6669	-	+	+	-	-	-	-	+	+	+	+	+	-	+	-	-	+	+	-	+	-	+	-	-	-
	GCA_002344205	*Opitutaceae* bacterium UBA2377	-	+	+	+	+	+	+	+	+	+	+	+	-	+	-	+	+	+	+	+	+	+	-	+	-

IMG ID	GenBank Accession	Taxon ID	infB	lepA	pheS	rplB	rplC	rplD	rplE	rplF	rplK	rplN	rplO	rplP	rpsB	rpsC	rpsE	rpsG	rpsH	rpsI	rpsJ	rpsK	rpsL	rpsM	rpsQ	rpsS	16S rRNA
	GCA_003142615	Fen-1467 sp003142615	-	+	+	+	+	+	+	+	+	+	+	+	-	+	+	+	+	+	+	+	+	+	-	+	+
	GCA_002310495	*Nibricoccus aquaticus*	-	+	+	+	+	+	+	+	+	+	+	+	-	+	+	+	+	+	+	+	+	+	-	+	+
	GCA_003096195	*Ereboglobus luteus* Ho45	-	+	+	+	+	-	+	+	+	+	+	+	-	+	-	+	+	+	+	+	+	+	-	+	+
	GCA_003641745	B12-G4 sp003641745	-	+	+	+	+	+	+	+	-	+	+	+	-	+	+	+	+	+	+	+	+	+	-	+	+
	GCA_002382525	IMCC26134 sp002382525	-	+	+	+	+	+	+	+	+	+	+	+	-	+	+	+	+	+	+	+	+	+	-	+	-
	GCA_003694825	J108 sp003694825	-	+	+	+	+	+	+	+	+	+	+	+	-	+	+	+	+	+	+	+	+	+	-	+	-
	GCA_003402695	Opi-474 sp003402695	+	+	+	+	+	+	+	+	+	+	+	+	-	+	-	+	+	+	+	+	+	+	-	+	-
	GCA_003151515	Palsa-1447 sp003151515	+	+	+	+	+	+	-	+	+	+	+	+	-	+	-	+	+	+	+	+	+	+	-	+	+
	GCA_002304445	Tous-C4FEB sp002304445	+	+	+	+	+	+	+	+	+	+	+	+	-	+	-	-	+	+	+	+	-	+	-	+	-
	JAABVE000000000	*Petrosia ficiformis*- associated bin0 (227c)	-	+	+	+	+	+	+	+	+	+	+	+	-	+	+	+	+	-	+	+	+	+	+	+	+
	JAABVD000000000	*Petrosia ficiformis*- associated bin01 (287ce)	+	+	+	+	+	+	+	+	+	+	+	+	-	+	+	+	+	-	+	+	+	+	+	+	+
	GCA_014529625.1	Strain F3	+	+	+	+	+	+	+	+	+	+	+	+	-	+	-	+	+	+	+	+	+	+	+	+	+
	GCA_014529675.1	Strain E3	+	+	+	+	+	+	+	+	+	+	+	+	+	+	+	+	+	+	+	+	+	+	+	+	+
	GCA_014529665.1	Strain A3	+	+	+	+	+	+	+	+	+	+	+	+	+	+	+	+	+	+	+	+	+	+	+	+	+
